# Single-cell landscape of lymphoid and myeloid heterogeneity across type-stratified craniofacial lesions in polyostotic fibrous dysplasia

**DOI:** 10.3389/fimmu.2026.1906418

**Published:** 2026-09-28

**Authors:** Hui-Chuan Duan, Xia Liang, De-Cheng Wang, Jie Yuan, Fan Yang

**Affiliations:** 1 Department of Plastic and Reconstructive Surgery, Shanghai 9th People’s Hospital, Shanghai Jiao Tong University School of Medicine, Shanghai, China; 2 Department of Addiction Medicine, Anding Hospital Wuhu Branch (Wuhu Fourth People’s Hospital), Wuhu, China; 3 The Research Center for Lin He Academician New Medicine, Institutes for Shanghai Pudong Decoding Life, Shanghai, China; 4 Lishui Key Laboratory of Brain Health and Severe Brain Disorders, Lishui Second People’s Hospital, Wenzhou Medical University, Lishui, China; 5 Bio-X Institutes, Key Laboratory for the Genetics of Developmental and Neuropsychiatric Disorders, Ministry of Education, Shanghai Jiao Tong University, Shanghai, China

**Keywords:** cell-cell communication, craniofacial lesions, histological subtypes, immune microenvironment, lymphoid cells, myeloid cells, polyostotic fibrous dysplasia, single-cell RNA sequencing

## Abstract

**Background:**

Polyostotic fibrous dysplasia (POFD) is a mosaic skeletal disorder driven by somatic *GNAS* mutations that constitutively activate Gsα-cAMP signaling, producing fibro-osseous craniofacial deformities. POFD lesions are histologically classified into three subtypes—Type I (connective-tissue predominant), Type II (cancellous-bone predominant), and Type III (sclerotic-bone predominant)—yet the immune microenvironment across these categories remains undefined. This study aims to dissect lymphoid and myeloid heterogeneity and inter-cellular communication networks among distinct POFD histological categories.

**Methods:**

We performed single-cell RNA sequencing on maxillary lesion tissues from three age- and sex-matched POFD patients classified as Type I (n=1), Type II (n=1), and Type III (n=1). After stringent quality control, 24,567 cells (median 1,762 genes/cell) were integrated and clustered. Ligand–receptor interactions were inferred using CellChat.

**Results:**

Four major lineages were resolved: mesenchyme, lymphoid, myeloid, endovascular. Lymphoid representation differed across subtypes (Type I 1.00%, Type II 5.10%, Type III 10.90%), whereas endovascular fractions showed an inverse pattern (Type I 25.10%, Type II 9.90%, Type III 4.50%). Sub-clustering identified five lymphoid and six myeloid subsets. CD4^+^ T cells were most abundant in Type I (51.10%) but less represented in Type II (36.50%) and Type III (29.30%). CD8^+^ T cells constituted 2.10%, 11.90%, and 11.20% across Types I-III, respectively; germinal-center (GC) B cells were undetectable in Type I, minor in Type II (~1.60%), and prominent in Type III (~29.20%). Among myeloid cells, M1 macrophages comprised 9.90% in Type I, 25.70% in Type II, and 15.30% in Type III; osteoclasts were abundant in Type I (35.40%) but scarce in Types II and III (<5%). Dendritic cells (DCs) were absent in Type I yet detectable in Type II (1.90%) and Type III (2.60%). Network-central genes included *LTB*, *TXNIP*, *FTH1*, and *PABPC1* for CD4^+^ and GC B cells, and *S100A8/9*, *CXCL8*, *CCL2/3/4*, and *IL1B* for M1 macrophages and monocytes. Inter-cellular communication networks differed markedly by subtype: Type I and II displayed relatively simple interactomes, whereas Type III exhibited densely interconnected signaling with strengthened M2 macrophage-monocyte, M2 macrophage-GC B cell (CXCL12-CXCR4, APP-CD74), and GC B cell-monocyte axes, alongside attenuated CD4^+^ T cell-GC B cell (CLEC2D-KLRB1, ADGRE5-CD55) and M1 macrophage-CD4^+^ T cell crosstalk. Pathway enrichment revealed subtype-specific activation of oxidative phosphorylation, NF-κB, and antigen-presentation cascades across lymphoid and myeloid compartments.

**Conclusions:**

This study provides the first single-cell immune atlas of POFD, delineating lymphoid and myeloid heterogeneity across three histological subtypes. The identified subtype-specific markers and inter-cellular communication architectures offer candidate biomarkers for diagnostic stratification and reveal potential immunomodulatory targets for this disfiguring bone disorder.

## 1 Introduction

McCune-Albright syndrome (MAS, MIM: 174800) is a prototypical mosaic disorder in which polyostotic fibrous dysplasia (POFD) represents a principal skeletal manifestation (1–4). MAS is characterized by post-zygotic GNAS mutations that constitutively activate Gs-coupled G protein-coupled receptors (GPCRs), thereby elevating intracellular cyclic AMP (cAMP) and driving downstream protein kinase A signaling that promotes osteoblast differentiation (5–9). Aberrant Gs-GPCR activation in bone produces expansile fibro-osseous lesions, craniofacial hyperostosis, and skeletal asymmetry (10, 11). Patients experience fragile bones, deformities, and pain. Beyond the skeleton, individuals with MAS display multisystem involvement, including cutaneous pigmentation and endocrine hyperfunction (12–14).

The skeleton undergoes continuous remodeling throughout life (15–18). Osteocytic communication governs metabolic homeostasis, coordinating bone formation and osteoclast-mediated resorption (19–23). Endothelial, stromal, and immune cells further modulate the bone microenvironment through complex interactions (24–32). B cells regulate osteoclastogenesis via RANKL expression (33–38), whereas resting T cells protect bone and activated T cells promote NF-κB and TNF-α production, driving osteoclast formation and bone loss (39–45). Monocytes support osteoblastic differentiation through BMP-2 and cytokine signaling (46–50). However, how lymphoid and myeloid subsets vary among POFD histological subtypes, which transcriptomic signatures distinguish these categories, and how their inter-cellular networks remodel the lesional microenvironment remain unclear—gaps that must be addressed to advance immune-informed diagnostics and therapeutics.

In this study, we applied single-cell RNA sequencing (scRNA-Seq) to characterize cellular heterogeneity and inter-cellular communication in maxillary lesions from three POFD patients classified as Type I (connective-tissue predominant), Type II (cancellous-bone predominant), and Type III (sclerotic-bone predominant). We identified distinct lymphocyte subtypes— including CD4^+^ and CD8^+^ T cells, germinal-center (GC) B cells, naïve T cells, and regulatory T cells (Treg)—and delineated their compositional and functional differences across subtypes. We also profiled myeloid populations, including M1 and M2 macrophages, monocytes, mast cells, osteoclasts, and dendritic cells, and identified key marker genes such as *LTB*, *TXNIP*, *FTH1*, and *PABPC1*. Furthermore, we mapped ligand-receptor interactomes and compared communication architectures among the three histological categories.

To our knowledge, this is the first single-cell study to systematically compare lymphoid and myeloid composition, function, and inter-cellular communication across distinct POFD histological subtypes. Our findings provide new insights into immune-mediated bone metabolic dysregulation and establish a foundation for identifying subtype-specific candidate diagnostic biomarkers and potential therapeutic targets in POFD.

## 2 Materials and methods

### 2.1 Subject recruitment

The study protocol was reviewed and approved by the Ethics Committee of Shanghai Ninth People’s Hospital, Shanghai Jiao Tong University School of Medicine (Approval number: SH9H-2024-TK193-1). Before any recruitment, all participants were fully informed of the study objectives and procedures, and they provided written informed consent. Three age- and sex-matched patients with distinct subtype of POFD were enrolled: (a) Type I POFD (connective tissue type): The lesion is replaced by gray-white fibrous connective tissue with focal hemorrhage and cystic degeneration; in severe cases, mucoid tissue is observed within the cyst wall. Fibrous connective tissue predominates over osseous elements, interspersed with small amounts of osteoid tissue and immature trabeculae that form tongue-shaped or petal-like bone ridges. In some lesions, intracystic liquefaction produces imaging features resembling bone cysts; the polycystic appearance results from overlapping bone ridges along cyst walls, manifesting as round or oval radiolucent areas of variable size. These lesions are sharply demarcated from adjacent normal bone, with a minority exhibiting foam-like (bubbly) changes; (b) Type II POFD (cancellous bone type): CT imaging demonstrates the classic ground-glass appearance, composed of newly formed immature primitive bone and delicate trabeculae. Normal trabecular architecture is effaced, yielding a homogeneous ground-glass pattern. In contrast to Type I, osseous tissue predominates, consisting predominantly of thin, thread-like or slender irregular trabeculae; and (c) Type III POFD (sclerotic bone type): CT imaging reveals a sclerotic or ground-glass appearance with thickened, tortuous trabeculae; dense, interconnected mature trabeculae predominate with minimal fibrous tissue, presenting as mass-like or nodular hyperostosis with uniformly increased bone density. Margins are well defined, and some lesions are surrounded by small foci of ground-glass change with sclerotic rims. Prior to surgery, all patients underwent comprehensive questionnaires, medical-history review, computed tomography (CT) examination, and physical examination. Specifically, clinical classification of POFD was established through multidisciplinary consensus and comprised the following components: (i) imaging assessment using CT with three-dimensional reconstruction to quantify lesion volume, mineral density (in Hounsfield units), and cortical bone integrity; (ii) clinical examination scoring facial asymmetry, swelling extent, and functional impairment (mastication, speech, and airway patency) according to the validated Craniofacial Fibrous Dysplasia Severity Score (CFDSS); and (iii) histopathological evaluation of intraoperative biopsy specimens by two independent pathologists blinded to clinical data, including grading of the fibrous-to-cartilaginous tissue ratio, cellularity, and vascularity. **Supplementary Figure 1** illustrates representative CT imaging result for each of the three POFD subtypes. All patients underwent resection of maxillary POFD lesion(s) and corrective facial surgery. Inclusion criteria were as follows: male, 35 years of age, first-time diagnosis of POFD, typical POFD lesions located in the maxilla; Exclusion criteria were: current or previous chronic disorders that could affect bone metabolism, including but not limited to diabetes mellitus, malabsorption syndromes, ankylosing spondylitis, thyroid or parathyroid disorders, malignancy, hepatic or renal failure, hematologic disorders, hyperprolactinemia, or pathological/traumatic fractures; history of smoking or alcohol use; history of substance dependence; medication use within the past year; or previous cosmetic facial surgery performed for non-medical indications.

### 2.2 Biospecimen collection

During surgery, maxillary POFD lesions were resected from identical anatomical sites. Specimens were rinsed with ice-cold 1× PBS, placed in sterile 2 mL tubes at 4 °C, and transported to the laboratory within 2 hours for downstream procedures.

### 2.3 Tissue dissociation and single-cell suspension

After removing adipose and necrotic tissue, samples were washed with ice-cold PBS. Tissue was minced to <1 mm^3^ pieces in dissociation solution (2.0 mg/mL type II collagenase; Sangon Biotech, Cat No. A004174-0001) and digested at 37 °C. Digestion was stopped with equal volume of resuspension solution (5% FBS + DMEM). The suspension was filtered through 70 µm and 40 µm cell strainers (Corning, Cat. No. 352350 & 352340) and centrifuged at 4 °C. Although mature osteoclasts were retained by the 40 µm strainer due to their large diameter (approximately 80-100 µm), mononuclear pre-osteoclasts (osteoclast precursors, approximately 10-20 µm) passed through and were recovered. Red blood cells were lysed using ACK-lysis buffer (ThermoFisher Scientific, Cat No. A1049201) for 5 minutes on ice. Debris and dead cells were removed using Miltenyi Debris Removal Solution (Cat No. 130-109-398) and Dead Cell Removal Kit (Cat No. 130-090-101), respectively. Cell concentration and viability were assessed by Countstar Rigel S2 after AO/PI staining.

### 2.4 Library preparation and high-throughput sequencing

Single-cell libraries were constructed using the DECODER 3’ Single-Cell Reagent Set (Dynamic Biosystems, Cat No.13010023) on the DECODER Microfluidic Isolation Platform (13030003-A1). cDNA was amplified, purified with solid-phase reversible immobilisation (SPRI) beads, and quantified by Qubit 4.0 (ThermoFisher, Q32854) and 4150 TapeStation System (Agilent, G2992AA). Indexed libraries were prepared per DECODER v1.0 protocol, verified by quantitative PCR, and sequenced on Illumina NovaSeq 6000 (150 bp paired-end) with ≥100 Gb raw data per sample.

### 2.5 Data pre-processing and quality control

Analyses were performed in R (v4.3.1) using Seurat (v4.4.0). Genes detected in <3 cells and cells with >20% mitochondrial reads, <300 or >7,500 unique molecular identifiers (UMIs), or <500 or >18,000 reads were excluded. Data integration used Harmony (RunHarmony), UMAP (RunUMAP), and FindNeighbors (50 dimensions). Clusters were annotated using established lineage markers (51–53). Final cell counts were: Type I lesions (mesenchyme 3,153; lymphoid 47; endovascular 1,141; myeloid 212), Type II lesions (8,307; 504; 979; 105), and Type III lesions (7,634; 1,098; 453; 934).

### 2.6 Batch-effect correction

Batch effects were diagnosed using scBatch (v1.2.0), scran (v1.24.0) (54), kBET (v0.99.5), and silhouette analysis (scikit-learn v1.1.2). Correction proceeded in two tiers: (i) Harmony (v0.1.1) (55) via Scanpy (v1.9.1) (56) (θ=2, max_iter=50, τ=0.8); (ii) fastMNN on 3,000 highly variable genes (HVGs) using scran (v1.24.0) (54) and BiocSingular (v1.12.0). Post-correction benchmarks: kBET rejection rate <5%, silhouette mean width <0.02, ARI ≥ 0.90. All downstream analyses used the dual-corrected dataset.

### 2.7 Dimensionality reduction and clustering

Feature selection retained 3,000 HVGs (Seurat v4.3.0). principal-component analysis (PCA), uniform manifold approximation and projection (UMAP, uwot v0.1.14; min_dist=0.3, spread=1.0, metric=cosine on 30 principal components) (55), and Leiden clustering (igraph v1.3.4) (57), were performed (resolution 0.6). Lymphoid and myeloid subsets were re-clustered using LIGER (v1.0.0; k=20, λ=5) at resolution 0.4. Doublets were detected by Scrublet (v0.2.3) (58) (6.2% expected rate); clusters with >20% doublets were re-evaluated. AUCell (v1.18.0) (59) verified marker robustness (mean AUC ≥0.65). Final annotations were validated against the Human Primary Cell Atlas and SingleR (v2.2.0) (60).

### 2.8 Functional enrichment analysis

Differential expression was tested between Type II versus Type I and Type III versus Type II within each population (Seurat FindMarkers; |log_2_FC| > 0.585, Bonferroni-corrected *P* < 0.01). Gene Ontology (GO) and Kyoto Encyclopedia of Genes and Genomes (KEGG) enrichment was performed via the Database for Annotation, Visualization and Integrated Discovery (DAVID). Subpopulations with <10 cellular barcodes per specimen were excluded.

### 2.9 Ligand-receptor interactome analysis

CellChat (v1.5.0) (61) inferred cell-cell communication using log-normalized counts (min. cellular barcodes=10, min. fractional threshold=0.1). Significance was determined by permutation (FDR q < 0.05); Network centrality (betweenness, information flow) ranked key interactions. Cross-subtype comparisons used CellChat’s differential-interaction module with default settings.

### 2.10 PPI network and hub-gene analysis

PPI network of DEGs was constructed using the Search Tool for the Retrieval of Interacting Genes (STRING, v11.5; confidence ≥0.7). Networks were visualized in Cytoscape (v3.9.1) (62) with cytoHubba extension (v2.0; MCC scoring). The top-ranking genes were designated as hubs. Functional relevance was validated via enrichR (v3.0) (63) against Gene Ontology-Biological Process (GO-BP) and KEGG.

### 2.11 Immunohistochemical validation

To independently validate the scRNA-seq findings, immunohistochemistry (IHC) was performed on independent formalin-fixed paraffin-embedded (FFPE) craniofacial lesional tissues from one patient per POFD subtype (Type I, Type II, and Type III). Following deparaffinization, rehydration, and heat-mediated antigen retrieval in citrate buffer (pH 6.0), 4 μm tissue sections were blocked with 5% normal goat serum and incubated overnight at 4 °C with primary antibodies against IL1B (1:800, Servicebio, GB11113). Signal detection was achieved using horseradish peroxidase (HRP)-conjugated secondary antibodies followed by 3,3’-diaminobenzidine (DAB) chromogen development, with hematoxylin counterstaining. Staining intensity (i.e. positive area) was analyzed and calculated by the Saiviewer software (Servicebio, V.3.0.1).

## 3 Results

### 3.1 Single-cell RNA sequencing reveals distinct cell types across POFD histological subtypes

To characterize cellular heterogeneity across distinct POFD histological categories, we performed scRNA-Seq on maxillary lesions from three patients classified as Type I (connective tissue type, n = 1), Type II (cancellous bone type, n = 1), and Type III (sclerotic bone type, n = 1) (**Figure 1A**). After dataset integration and stringent quality control, we obtained 24,567 cells (mean 1,762 genes/cell) (**Figure 1B**). Cells partitioned into four major lineages: mesenchyme, lymphoid, endovascular, and myeloid (**Figure 1C**), which resolved into 18 transcriptionally distinct clusters (**Figure 1D**). Proportional analysis showed mesenchyme as the dominant fraction (Type I 69.30%, Type II 84.00%, Type III 75.40%), followed by endovascular (Type I 25.10%, Type II 9.90%, Type III 4.50%), lymphoid (Type I 1.00%, Type II 5.10%, Type III 10.90%), and myeloid (Type I 4.70%, Type II 1.10%, Type III 9.20%) subsets (**Figure 1E**). These subtype-associated compositional differences suggest that lymphoid and endovascular compartments vary with lesional histology.

**Figure 1:**
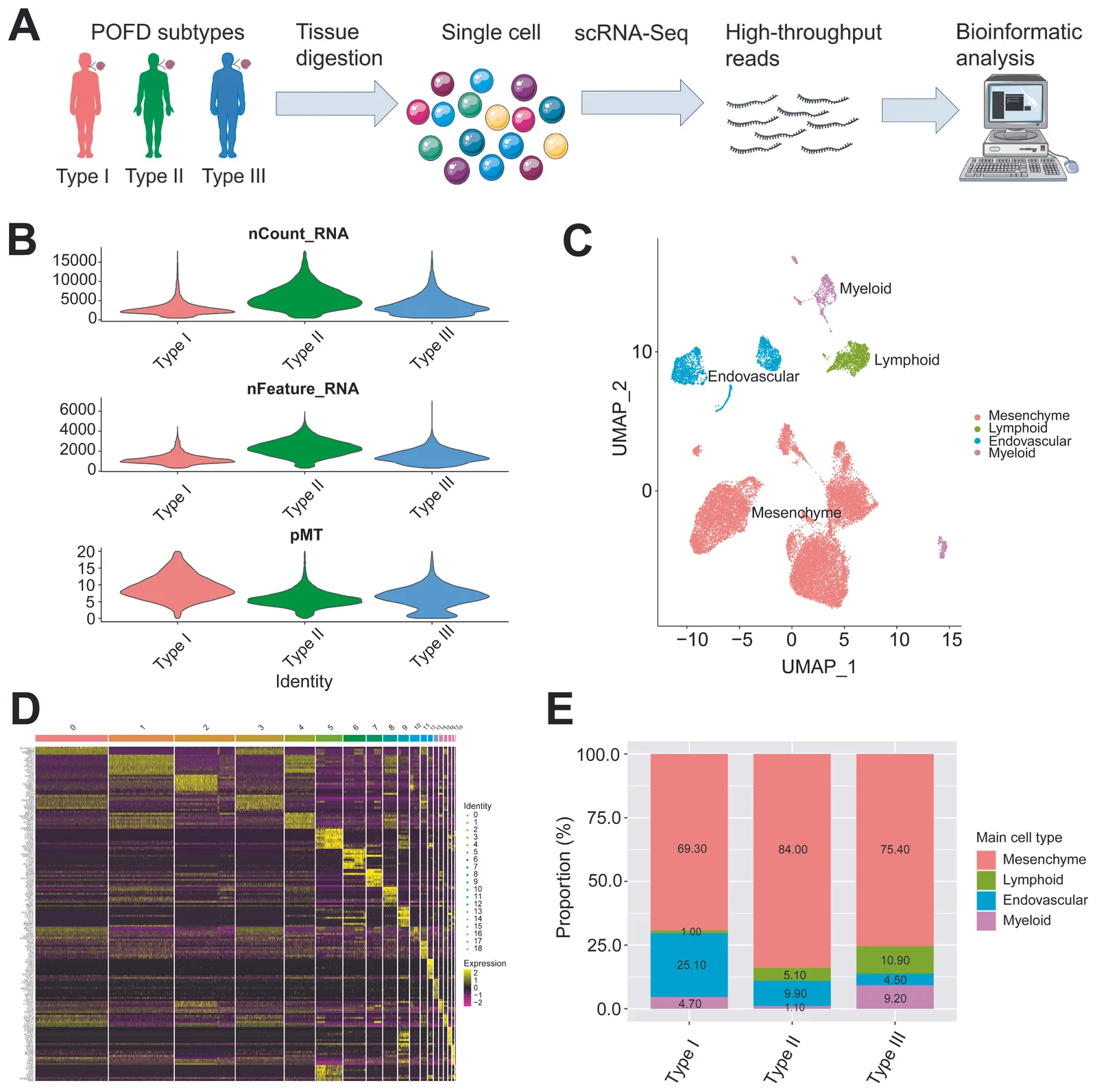
scRNA-Seq reveals four major lineages in different POFD subtypes. **(A)** Workflow of this study. POFD subtypes: Type I, Type II, and Type III. **(B)** Violin plot shows the number of unique molecular identifiers (UMI) per cell (upper panel), genes per cell (middle panel), and the percentage of mitochondrial RNAs in each sample, after rigorous QC. **(C)** t-SNE plot presents the clustering of four major lineages, including mesenchyme, lymphoid, myeloid, and endovascular, in POFD lesions. **(D)** Heatmap shows the top-ranking genes with the highest avg_logFC of each cluster. **(E)** Bar plot demonstrates the proportion of each population (i.e., mesenchyme, lymphoid, myeloid, and endovascular) in the three histological subtypes of POFD.

### 3.2 Functional analyses and identification of hub genes for DEGs of lymphoid and myeloid lineage cells

To identify hub genes, we performed protein-protein interaction (PPI) analyses on lymphoid and myeloid differentially expressed genes (DEGs). In Type II lymphoid cells, the top-50 hub genes included *HSPA1B* (a stress-inducible heat-shock protein chaperone), *HNRNPA1* (an RNA-binding protein regulating mRNA splicing and stability), *EEF2* (eukaryotic translation elongation factor 2), *RACK1* (receptor for activated C kinase 1), *CALM2* (a calcium-binding signal transducer), and *ACTG1* (a cytoskeletal actin isoform) relative to Type I (**Figure 2A**). Type III lymphoid cells showed a distinct hub set comprising *HSPA6* (an ATP-dependent heat-shock protein chaperone), *HSPE1* (heat-shock protein family E member 1), *HSPA1B*, *HNRNPA1*, *EIF4A1* (eukaryotic translation initiation factor 4A1), and *PABPC1* (poly(A)-binding protein cytoplasmic 1, an mRNA-stability regulator) compared with Type II (**Figure 2B**). Type II myeloid cells displayed hub genes such as *CXCR2*, *CCL3L1*, *FCGR3B*, *MMP9*, *ANXA2* (annexin A2), and *CTSK* (cathepsin K) versus Type I (**Figure 2C**), whereas Type III myeloid cells exhibited a unique signature including *CCL2*, *CCL3*, *CCL4*, *CXCL2*, *CXCL3*, *CXCL8*, and *CCR7* relative to Type II (**Figure 2D**).

**Figure 2:**
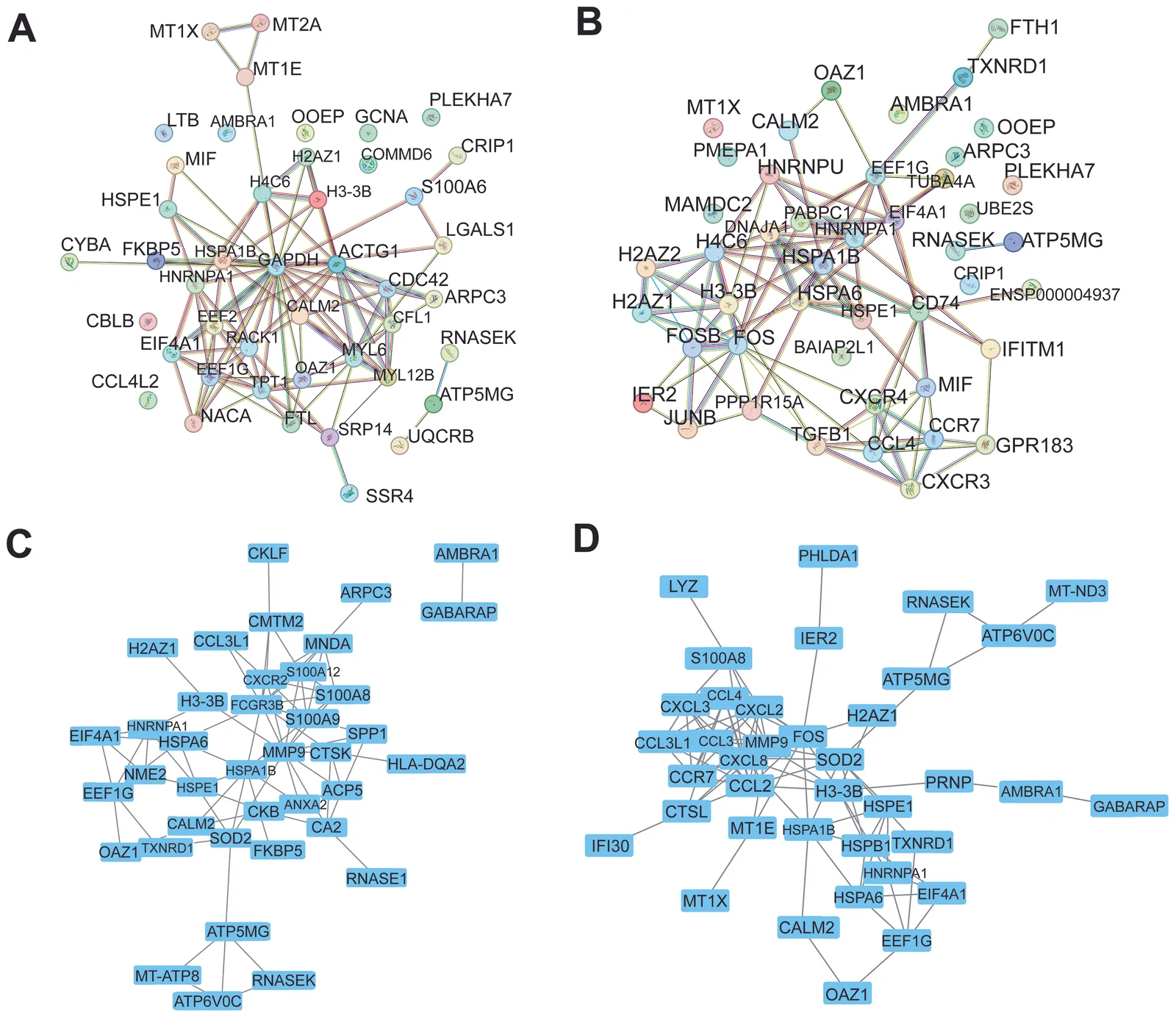
Protein-protein interaction networks of lymphoid and myeloid DEGs. **(A, B)** Protein-protein interaction (PPI) networks of top-50 DEGs of lymphoid in Type II versus Type I, and Type III versus Type II lesions, respectively. **(C, D)** PPI networks of top-50 DEGs of myeloid in Type II versus Type I, and Type III versus Type II lesions, respectively.

### 3.3 Identification of lymphoid and myeloid subsets and their marker genes

To compare lymphoid and myeloid subsets distributions among POFD histological subtypes, we isolated 1,649 lymphoid cells for sub-clustering. Five lymphoid subtypes were identified: CD4^+^ T cells, CD8^+^ T cells, naïve T cells, GC B cells, and Tregs (**Figure 3A**). Compositional analysis revealed marked differences across subtypes. CD4^+^ T cells and naïve T cells predominated in Type I (95.80%) and Type II (86.10%) lesions (**Figure 3B**). CD4^+^ T-cell abundance decreased from Type I (51.10%) to Type II (36.50%) and Type III (29.30%) (**Figure 3B**). CD8^+^ T cells constituted 2.10% in Type I, 11.90% in Type II, and 11.20% in Type III (**Figure 3B**). GC B cells were absent in Type I, minor in Type II (~1.60%), and prominent in Type III (~29.20%) (**Figure 3B**). These patterns implicate CD4^+^ and CD8^+^ T cells, together with GC B cells, as key immune constituents that differ across POFD histological categories.

**Figure 3:**
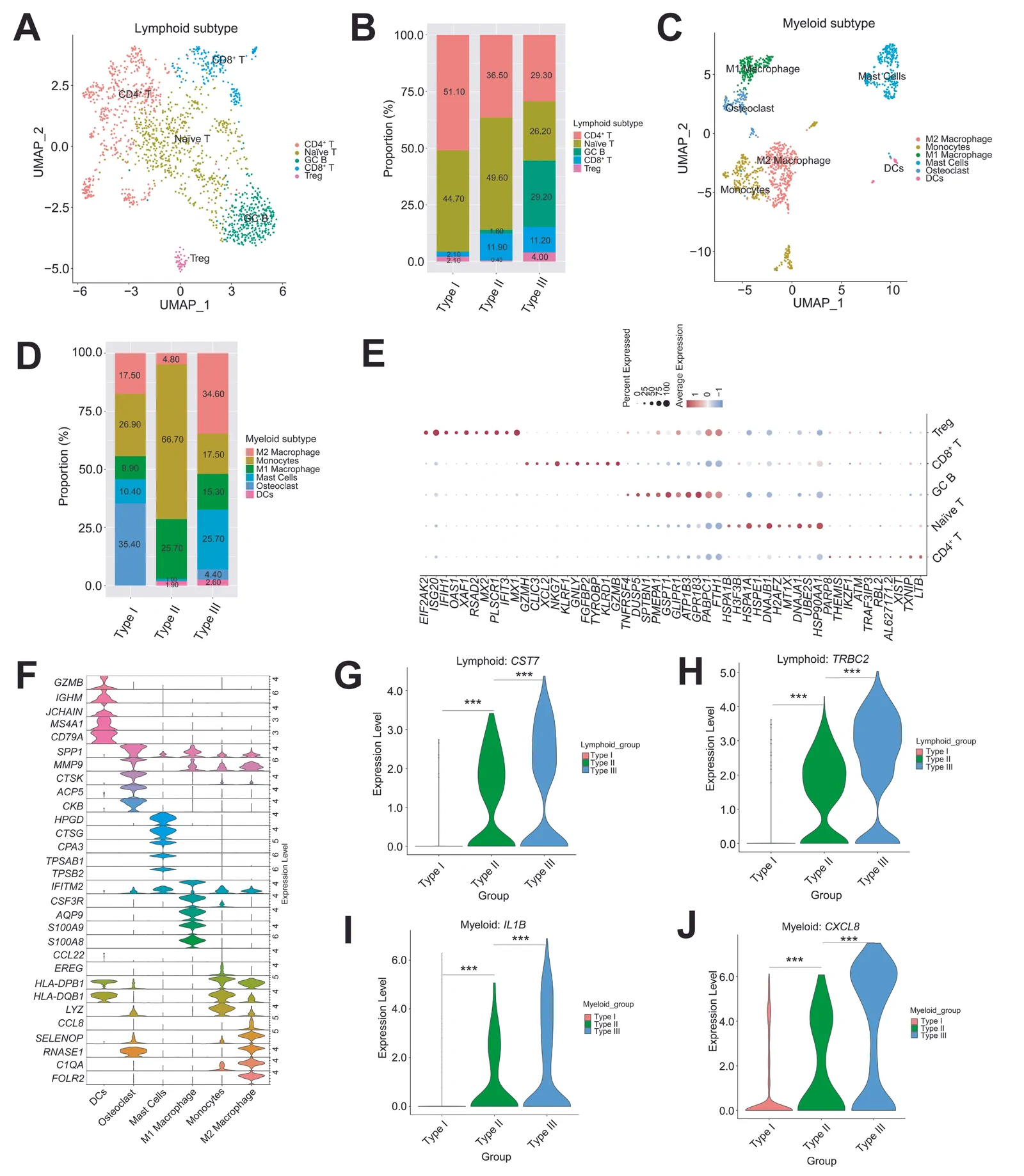
Sub-clustering analysis identifies five lymphoid and six myeloid subsets. **(A)** t-SNE plot shows the color-coded clustering of five lymphoid subsets, including CD4^+^ T cells, CD8^+^ T cells, GC B cells, Naïve T cells, and Tregs. **(B)** Bar plot shows the proportion of each subset in Type I, Type II, and Type III lesions. **(C)** t-SNE plot presents the color-coded clustering of six myeloid subsets, including M1 macrophages, M2 macrophages, monocyte, mast cells, DCs, and osteoclasts. **(D)** Bar plot shows the percentage of each subset in Type I, Type II, and Type III lesions. **(E)** Bubble plot shows the expression levels of ten representative marker genes for CD4^+^ T cells, Naïve T cells, GC B cells, CD8^+^ T cells, and Tregs. **(F)** Violin plot shows the expression levels of five representative marker genes for DCs, osteoclasts, mast cells, M1 macrophages, monocytes, and M2 macrophages. **(G, H)** Violin plot presents the increasingly expression levels of *CST7* and *TRBC2* gene of lymphoid in Type I, Type II, and Type III lesions, respectively. **(I)** and **(J)** Violin plot shows the increasingly expression levels of *IL1B* and *CXCL8* gene of myeloid in Type I, Type II, and Type III lesions, respectively. Group comparisons were performed using independent-samples t-tests. ***P < 0.001.

We also identified six myeloid subsets among 1,251 cells: M1 and M2 macrophages, monocytes, mast cells, osteoclasts, and dendritic cells (DCs) (**Figure 3C**). Myeloid composition differed markedly by subtype. In Type II, M1 macrophages and monocytes comprised 92.40% of the compartment (**Figure 3D**). M1 macrophages were elevated in Type II (25.70%) and Type III (15.30%) compared with Type I (9.90%) (**Figure 3D**). Osteoclasts constituted 35.40% of myeloid cells in Type I, yet were scarce in Type II (1.00%) and Type III (4.40%) (**Figure 3D**). Conversely, DCs were absent in Type I, present at 1.90% in Type II, and 2.60% in Type III (**Figure 3D**). These findings indicate that M1 macrophages, osteoclasts, and DCs are key myeloid constituents that vary across POFD subtypes.

We next ranked top discriminative genes for each subset. Among lymphoid cells, Tregs expressed *EIF2AK2*, *ISG20*, *IFIH1*, *OAS1*, and *XAF1*; CD8^+^ T cells expressed *GZMH*, *CLIC3*, *XCL2*, *NKG7*, and *KLRF1*; GC B cells expressed *FTH1*, *PABPC1*, *GPR183*, *ATP1B3*, and *GLIPR1*; naïve T cells expressed *HSP90AA1*, *UBE2S*, *DNAJA1*, *MT1X*, and *DNAJB1*; and CD4^+^ T cells expressed *LTB*, *TXNIP*, *XIST*, *RBL2*, and *TRAF3IP3* (**Figure 3E**). Among myeloid subsets, DCs expressed *GZMB*, *IGHM*, *JCHAIN*, *MS4A1*, and *CD79A*; osteoclasts expressed *SPP1*, *MMP9*, *CTSK*, *ACP5*, and *CKB*; mast cells expressed *HPGD*, *CTSG*, *CPA3*, *TPSAB1*, and *TPSB2*; M1 macrophages expressed *IFITM2*, *CSF3R*, *AQP9*, *S100A9*, and *S100A8*; M2 macrophages expressed *CCL8*, *SELENOP*, *RNASE1*, *C1QA*, and *FOLR2*; and monocytes expressed *EREG*, *HLA-DPB1*, *HLA-DQB1*, and *LYZ* (**Figure 3F**). *CST7* and *TRBC2* expression in lymphoid cells was lowest in Type I and highest in Type III (*P* < 0.05; **Figures 3G, H**). *IL1B* and *CXCL8* expression in myeloid cells was elevated in Type II and Type III relative to Type I (*P* < 0.05; **Figures 3I, J**). IHC validation revealed that the IL1B expression trend across Type I, Type II, and Type III lesions was consistent with scRNA-seq data (**Supplementary Figures 2A–D**). These results indicate that the differential expression of subpopulation marker genes (e.g., IL1B) among the three POFD subtypes may reflect genuine biological variation, although further validation in larger independent cohorts is warranted.

Feature plots confirmed marker expression across lymphoid and myeloid subsets. CD4^+^ T cells expressed *LTB* and *AL627171.2*; CD8^+^ T cells expressed *GZMB* and *GNLY*; naïve T cells expressed *DNAJA1* and *MT1X*; Tregs expressed *MX1* and *XAF1*; and GC B cells expressed *FTH1* and *TNFRSF4* (**Figures 4A–E**). M2 macrophages expressed *RNASE1* and *SELENOP*; mast cells expressed *TPSB2* and *TPSAB1*; monocytes expressed *LYZ* and *EREG*; osteoclasts expressed *ACP5* and *CTSK*; M1 macrophages expressed *S100A8* and *S100A9*; and DCs expressed *JCHAIN* and *IGHM* (**Figures 4 F–K**). Furthermore, we identified lymphoid and myeloid subset-specific genes. For instance, GC B cells uniquely expressed *IL4R* among lymphoid subsets (**Figure 4L**), and osteoclasts exclusively expressed *ACP5* among myeloid subsets (**Figure 4M**).

**Figure 4:**
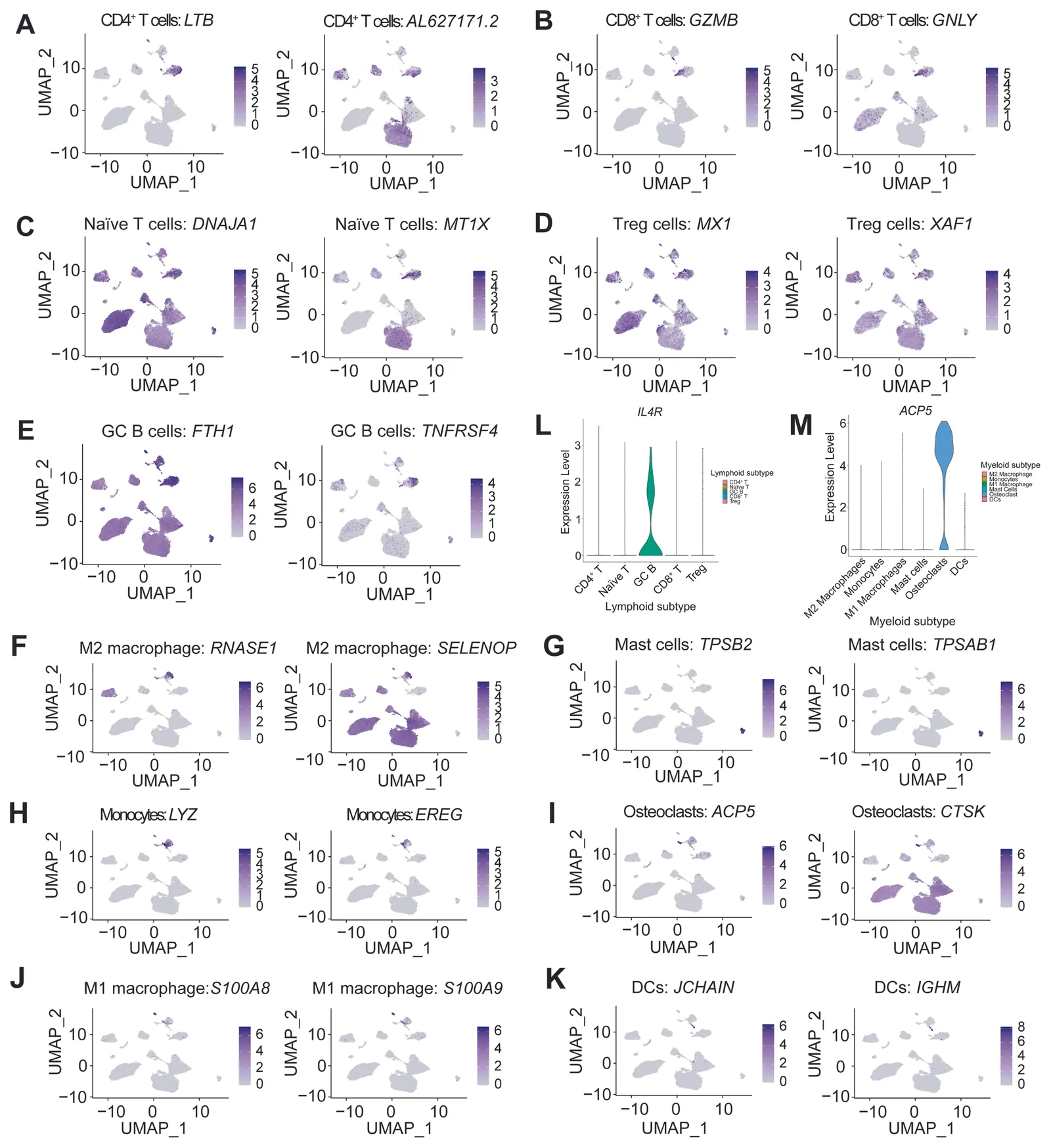
Representative signature genes for different lymphoid and myeloid subsets. **(A–E)** t-SNE dimension reduction map shows the signature genes for CD4^+^ T cells (*LTB* and *AL627171.2*), CD8^+^ T cells (*GZMB* and *GNLY*), Naïve T cells (*DNAJA1* and *MT1X*), Tregs (*MX1* and *XAF1*), and GC B cells (*FTH1* and *TNFRSF4*), respectively. **(F–K)** t-SNE dimension reduction map demonstrates the signature genes for M2 macrophages (*RNASE1* and *SELENOP*), mast cells (*TPSB2* and *TPSAB1*), monocytes (*LYZ* and *EREG*), osteoclasts (*ACP5* and *CTSK*), M1 macrophages (*S100A8* and *S100A9*), and DCs (*JCHAIN* and *IGHM*), respectively. **(L, M)** Violin plot shows the expression level of marker gene *IL4R* and *ACP5* in different lymphoid and myeloid subsets, respectively.

### 3.4 Pathway-enrichment analysis of DEGs across distinct T-cell subsets

To identify signaling pathways engaged by T-cell DEGs, we performed GO and KEGG enrichment analyses. In Type II versus Type I, CD4^+^ T-cell DEGs were enriched for cytoplasmic translation, ribosomal small-subunit biogenesis and assembly, ncRNA/rRNA processing and metabolism, and SSU-rRNA maturation. Cellular components included the ribosome, focal adhesion, cell-substrate junction, small-subunit processome, and polysome (**Figure 5A**). Molecular functions encompassed structural ribosomal constituents, rRNA binding, unfolded protein binding, translation factor/regulator activity, calcium-dependent protein binding, and mRNA 5′-UTR binding (**Figure 5A**). KEGG analysis showed over-representation of leukocyte transendothelial migration, phagosome, ribosome, and neutrophil extracellular trap formation pathways (**Figure 5B**).

**Figure 5:**
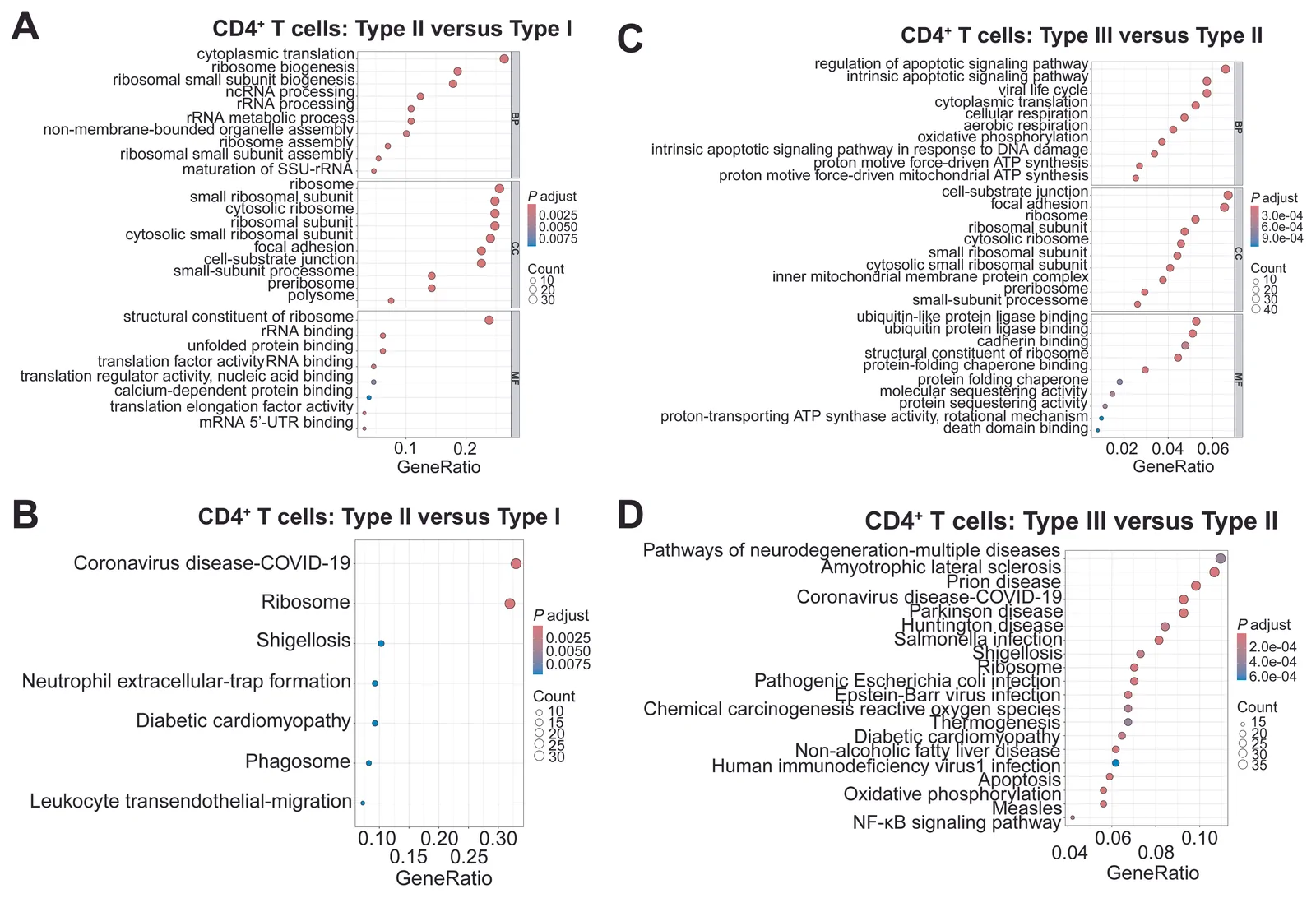
Functional and pathway enrichment analysis of DEGs in CD4^+^ T cells. **(A)** Gene ontology (GO) enrichment analysis of CD4^+^ T cells DEGs in Type II versus Type I POFD. **(B)** KEGG pathway enrichment analysis of CD4^+^ T cells DEGs in Type II versus Type I POFD. **(C)** GO enrichment analysis of CD4^+^ T cells DEGs in Type III versus Type II lesions. **(D)** KEGG pathway enrichment analysis of CD4^+^ T cells DEGs in Type III versus Type II lesions. **(A, C)** BP, biological process; CC, cellular compartment; MF, molecular function.

In Type III versus Type II, CD4^+^ T-cell DEGs were enriched for apoptotic signaling regulation, viral life cycle, cytoplasmic translation, aerobic respiration, oxidative phosphorylation, and mitochondrial ATP synthesis. Cellular components included cell-substrate junction, focal adhesion, ribosome, inner mitochondrial membrane protein complex, and small-subunit processome (**Figure 5C**). Molecular functions included ubiquitin ligase binding, cadherin binding, ribosomal constituents, protein-folding chaperone binding, and ATP synthase activity (**Figure 5C**). KEGG pathways included NF-κB signaling, oxidative phosphorylation, apoptosis, and neurodegeneration (**Figure 5D**). These data indicate that CD4^+^ T-cell transcriptomic profiles differ substantially between Type II and Type III.

CD8^+^ T-cell DEGs in Type III versus Type II were enriched for immune response, cytoplasmic translation, cell killing, ribosomal biogenesis, leukocyte-mediated cytotoxicity, and ATP synthesis. Cellular compartments included the ribosome, ficolin-1-rich granule, preribosome, inner mitochondrial membrane protein complex, processome, and cytolytic granule (**Supplementary Figure 3A**). Molecular functions encompassed ribosomal constituents, transcription factor binding, ubiquitin ligase binding, immune receptor activity, nucleosomal DNA binding, and ATP synthase activity (**Supplementary Figure 3A**). KEGG pathways included oxidative phosphorylation, apoptosis, lipid metabolism, NK-cell cytotoxicity, and ROS-related chemical carcinogenesis (**Supplementary Figure 3B**). These findings demonstrate distinct CD8^+^ T-cell transcriptomic profiles between Type II and Type III.

Naïve T-cell DEGs in Type II versus Type I showed overlapping GO and KEGG signatures with CD4^+^ T-cell findings (**Supplementary Figures 4A, B**). Similarly, naïve T-cell DEGs in Type III versus Type II recapitulated these patterns (**Supplementary Figures 4C, D**), suggesting that CD4^+^ and naïve T-cell transcriptomic changes are concordant across POFD subtypes.

### 3.5 Pathway-enrichment analysis of DEGs across distinct myeloid subsets

To characterize myeloid signaling pathways, we performed GO and KEGG enrichment on DEGs from M1 macrophages, monocytes, and osteoclasts. In Type II versus Type I, M1-macrophage DEGs were enriched for energy derivation, aerobic respiration, oxidative phosphorylation, and ATP synthesis. Cellular compartments included the mitochondrial inner membrane, respirasome, oxidoreductase complex, respiratory chain complex, and NADH dehydrogenase complex (**Figure 6A**). Molecular functions included transmembrane transporter, electron transfer, and NADH dehydrogenase activities (**Figure 6A**). KEGG pathways included proteasome, TCA cycle, phagosome, lysosome, retrograde endocannabinoid signaling, protein processing, ROS-related chemical carcinogenesis, and oxidative phosphorylation (**Figure 6B**). These findings indicate distinct M1-macrophage transcriptomic profiles between Type I and Type II.

**Figure 6:**
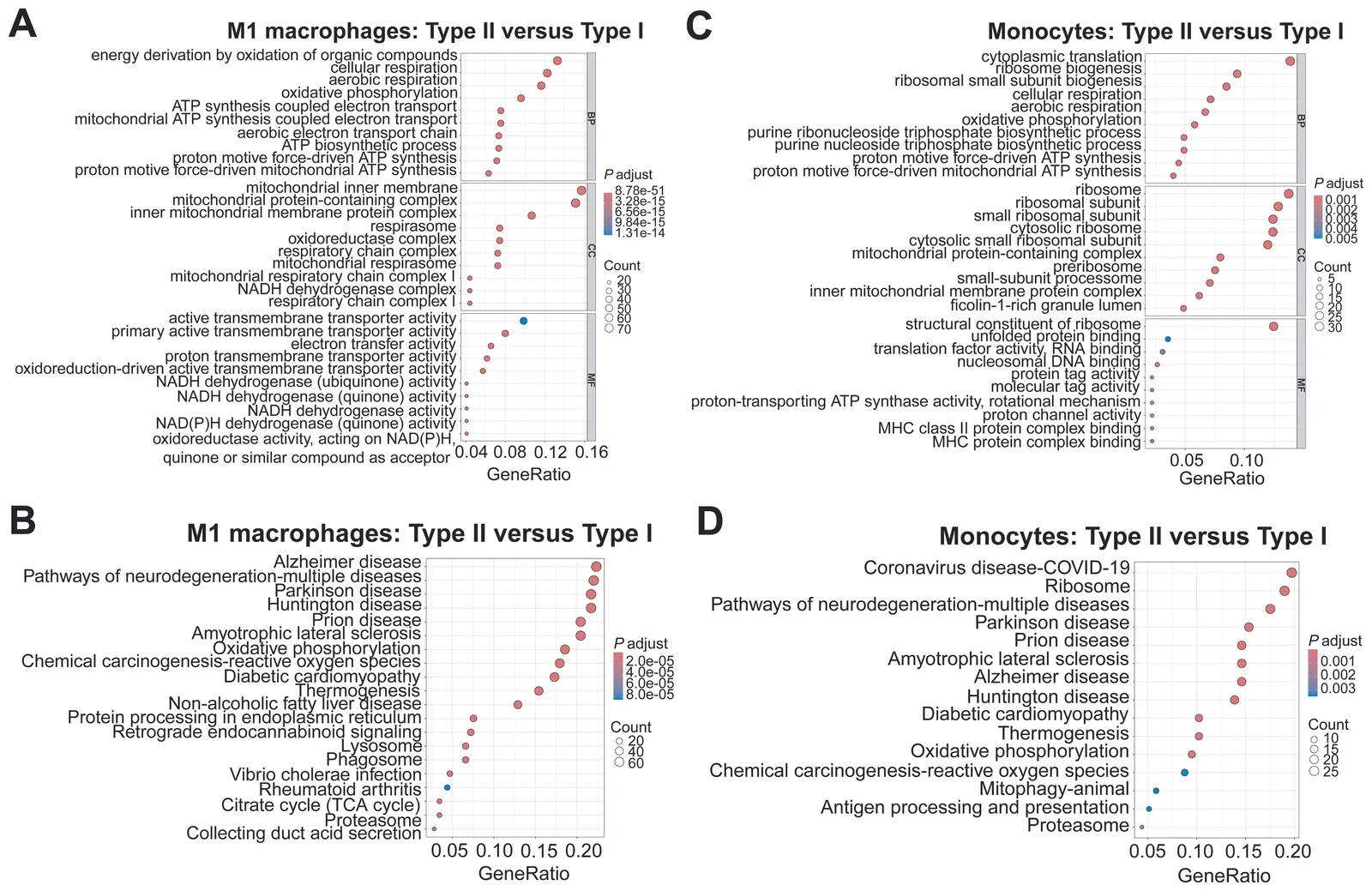
Functional and pathway enrichment analysis of DEGs in M1 macrophages and monocytes. **(A)** GO enrichment analysis of M1 macrophages DEGs in Type II versus Type I lesions. **(B)** KEGG pathway enrichment analysis of M1 macrophages DEGs in Type II versus Type I lesions. **(C)** GO enrichment analysis of monocytes DEGs in Type II versus Type I lesions. **(D)** KEGG pathway enrichment analysis of monocytes DEGs in Type II versus Type I lesions. **(A, C)** BP, biological process; CC, cellular compartment; MF, molecular function.

Monocyte DEGs in Type II versus Type I were enriched for cytoplasmic translation, ribosome biogenesis, aerobic respiration, oxidative phosphorylation, and ATP synthesis. Cellular compartments included ribosome, small ribosomal subunit, preribosome, inner mitochondrial membrane, and ficolin-1-rich granule (**Figure 6C**). Molecular functions included ribosomal constituents, unfolded protein binding, translation factor activity, RNA binding, ATP synthase activity, rotational-mechanism proton-channel activity, and MHC class II protein complex binding (**Figure 6C**). KEGG pathways included proteasome, antigen processing, mitophagy, ROS-related chemical carcinogenesis, oxidative phosphorylation, and neurodegeneration (**Figure 6D**). In Type III versus Type II, monocyte DEGs were enriched for apoptotic signaling regulation, lymphocyte activation, cytoplasmic translation, antigen processing, and peptide presentation via MHC class II. Cellular compartments included focal adhesion, cell-substrate junction, lysosomal membrane, ribosome, and cytoplasmic vesicle lumen (**Supplementary Figure 5A**). Molecular functions included transcription factor binding, ubiquitin ligase binding, cadherin binding, MHC protein complex binding, and miRNA inhibitor activity (**Supplementary Figure 5A**). KEGG pathways included lipid metabolism, ROS-related chemical carcinogenesis, and ribosome (**Supplementary Figure 5B**). These data demonstrate subtype-specific monocyte transcriptomic profiles.

Osteoclast DEGs in Type III versus Type I were enriched for cell-cell adhesion regulation, leukocyte activation, antigen processing, and MHC class II presentation. Cellular compartments included endocytic vesicle, ER-to-Golgi transport vesicle, endocytic vesicle membrane, and MHC class II complex (**Supplementary Figure 6A**). Molecular functions encompassed peptide antigen binding, GPCR binding, MHC class II complex binding, cytokine and chemokine activity, and MHC class II receptor activity (**Supplementary Figure 6A**). KEGG pathways included Th17 differentiation, antigen processing, Toll-like receptor signaling, phagosome, Th1/Th2 differentiation, and IL-17 signaling (**Supplementary Figure 6B**).

### 3.6 Intercellular communication networks across POFD histological subtypes

To dissect intercellular communication architectures, we mapped ligand-receptor interactions among lymphoid and myeloid subsets across POFD subtypes. In Type I and Type II lesions, extensive crosstalk occurred among M1 macrophages, M2 macrophages, monocytes, CD4^+^ T cells, and naïve T cells (**Figures 7A, B**). In Type II, M1-M2 macrophage, M1 macrophage-monocyte, and M2 macrophage-monocyte interactions were strengthened compared with Type I, whereas M1 macrophage-CD4^+^ T cell, M1 macrophage-naïve T cell, and naïve-CD4^+^ T cell communications were attenuated (**Figures 7A, B**). In Type III, the interactome became more complex, incorporating CD8^+^ T cells, GC B cells, and Tregs (**Figures 7C, D**). M2 macrophage-monocyte, M2 macrophage-GC B cell, and GC B cell-monocyte interactions were enhanced in Type III versus Type II, whereas M1 macrophage-CD4^+^ T cell, GC B-CD4^+^ T cell, and Treg-CD4^+^ T cell crosstalk diminished (**Figures 7C, D**).

**Figure 7:**
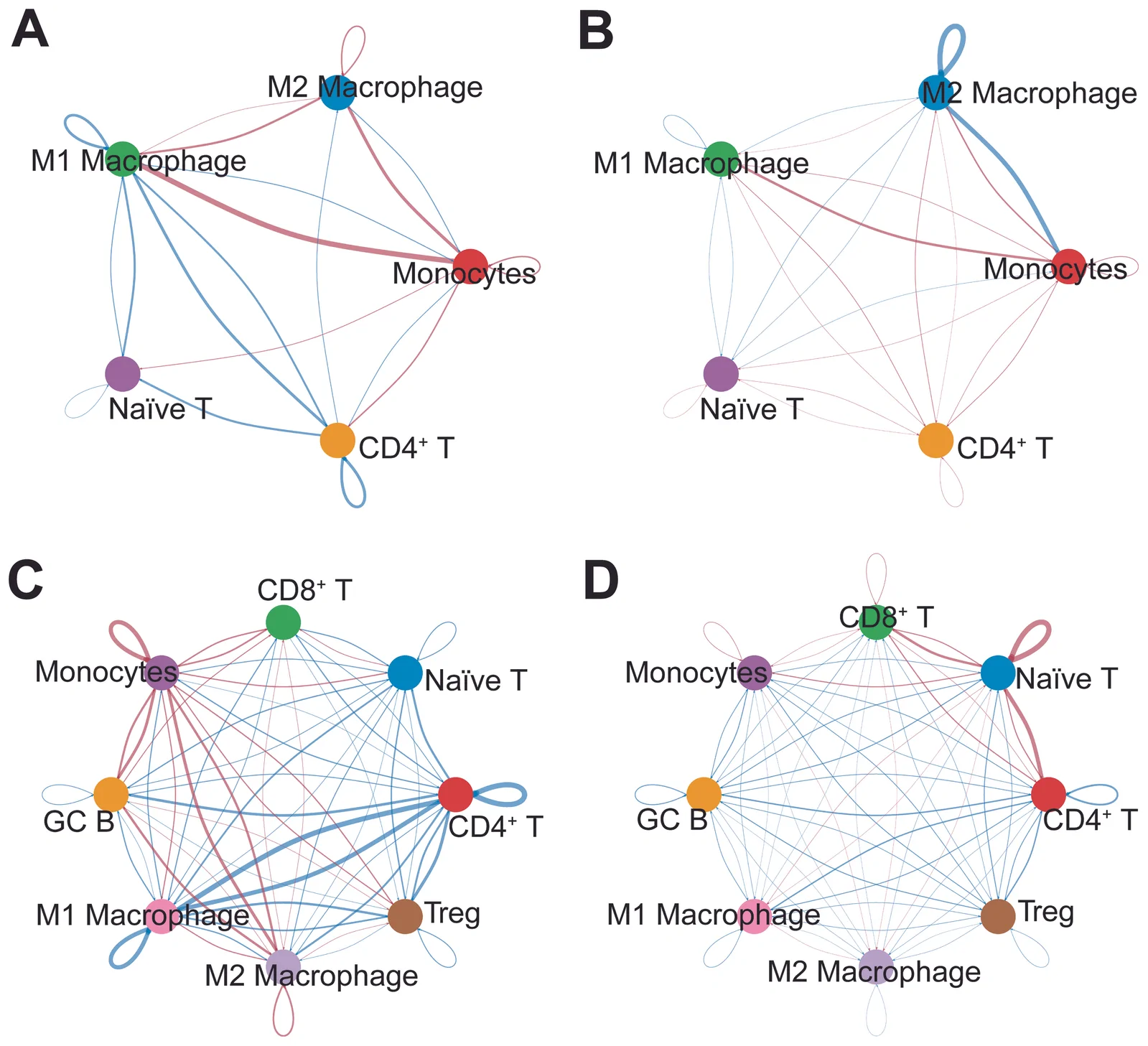
Cellular communication networks between distinct lymphoid and myeloid subsets in the three histological subtypes of POFD. **(A, B)** Cell-cell interaction [**(A)** left] and strength [**(B)** right] comparison between the Type II and Type I lesions, red and blue lines mean interaction number in Type II lesions was larger and smaller, respectively. **(C, D)** Cell-cell interaction [**(C)**, left] and strength [**(D)** right] comparison between the Type III and Type II lesions, red and blue lines mean interaction number in Type III lesions was larger and smaller, respectively.

In Type I lesions, ITGB2-ICAM1 and PTPRC-MRC1 mediated M2 macrophage-monocyte communication, whereas TNF-TNFRSF1B and PTPRC-MRC1 contributed to naïve T cell-monocyte crosstalk (**Figure 8**). PTPRC-MRC1 also facilitated CD4^+^ T cell-monocyte exchange in Type I (**Figure 8**). In Type II lesions, CD74-CXCR4 and CD74-CD44 shaped naïve T cell-monocyte and CD4^+^ T cell-monocyte communication, respectively (**Figure 8**).

**Figure 8:**
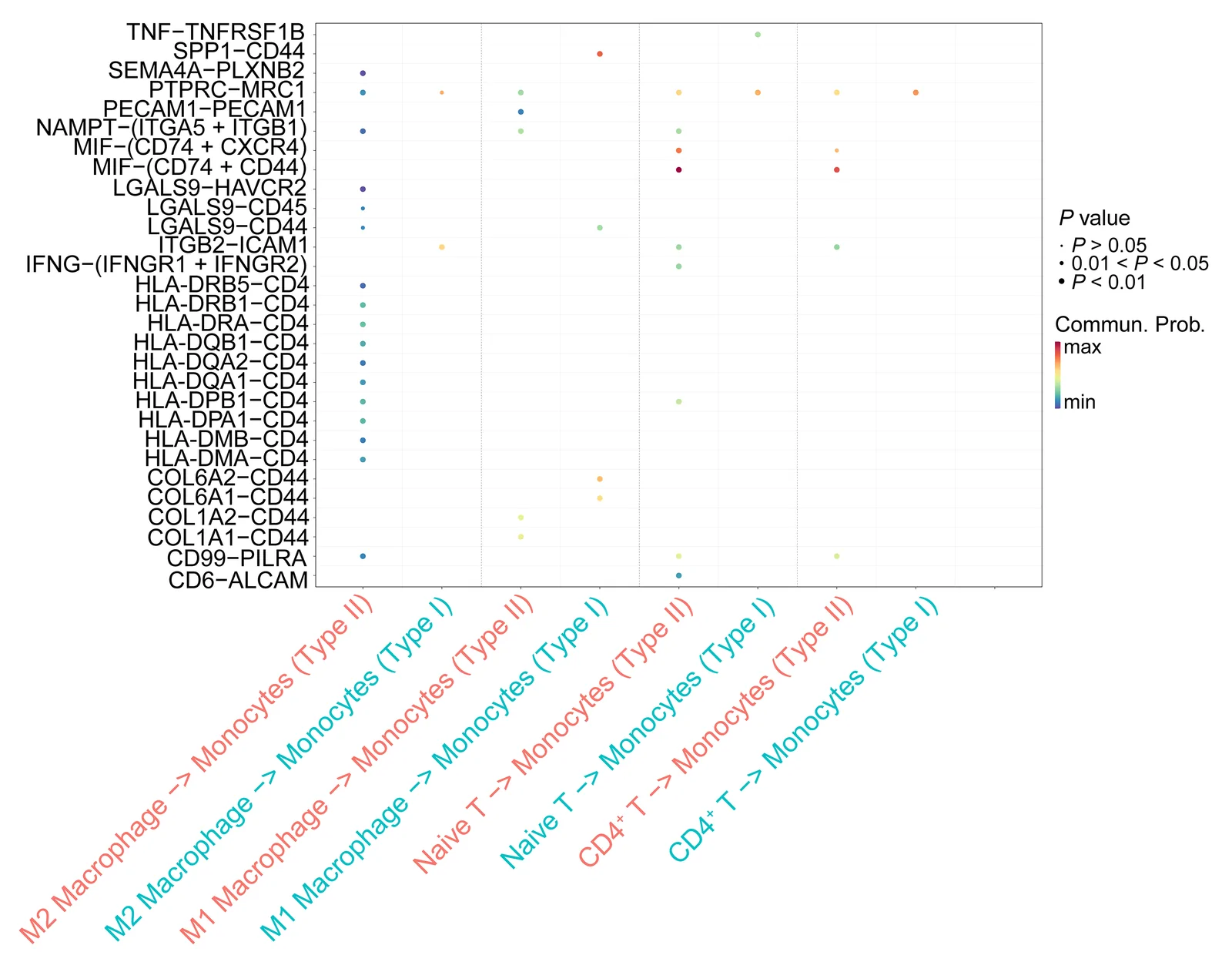
Ligand-receptor interactions between different lymphoid and myeloid subsets in Type II versus Type I POFD. Overview of representative ligand-receptor interactions between different subsets of lymphoid (CD4^+^ and CD8^+^ T cell, Naïve T cell, GC B cell, and Tregs) and myeloid (M1 and M2 type macrophage, monocytes, mast cells, DCs, and osteoclast) in Type II versus Type I POFD lesions.

In Type III POFD, CD99-PILRA, CD6-ALCAM, and ADGRE5-CD55 modulated CD4^+^ T cell-monocyte communication (**Figure 9**). CLEC2B/C/D-KLRB1 mediated CD4^+^ T cell-GC B cell and naïve T cell-GC B cell crosstalk (**Figure 9**). ADGRE5-CD55 and CLEC2B/C/D-KLRB1 also contributed to CD8^+^ T cell-GC B cell, CD8^+^ T cell-Treg, and naïve T cell-Treg interactions (**Figure 9**). SPP1-CD44, COL1A1-CD44, and COL1A2-CD44 underpinned M1 macrophage-monocyte, M1 macrophage-GC B cell, and M1 macrophage-Treg communications (**Figure 9**). Conversely, TNF-TNFRSF1B, SPP1-CD44/CD45, LGALS9-CD45, CXCL12-CXCR4, and APP-CD74 shaped M2 macrophage-monocyte, M2 macrophage-GC B cell, and M2 macrophage-Treg signaling in Type III lesions (**Figure 9**).

**Figure 9:**
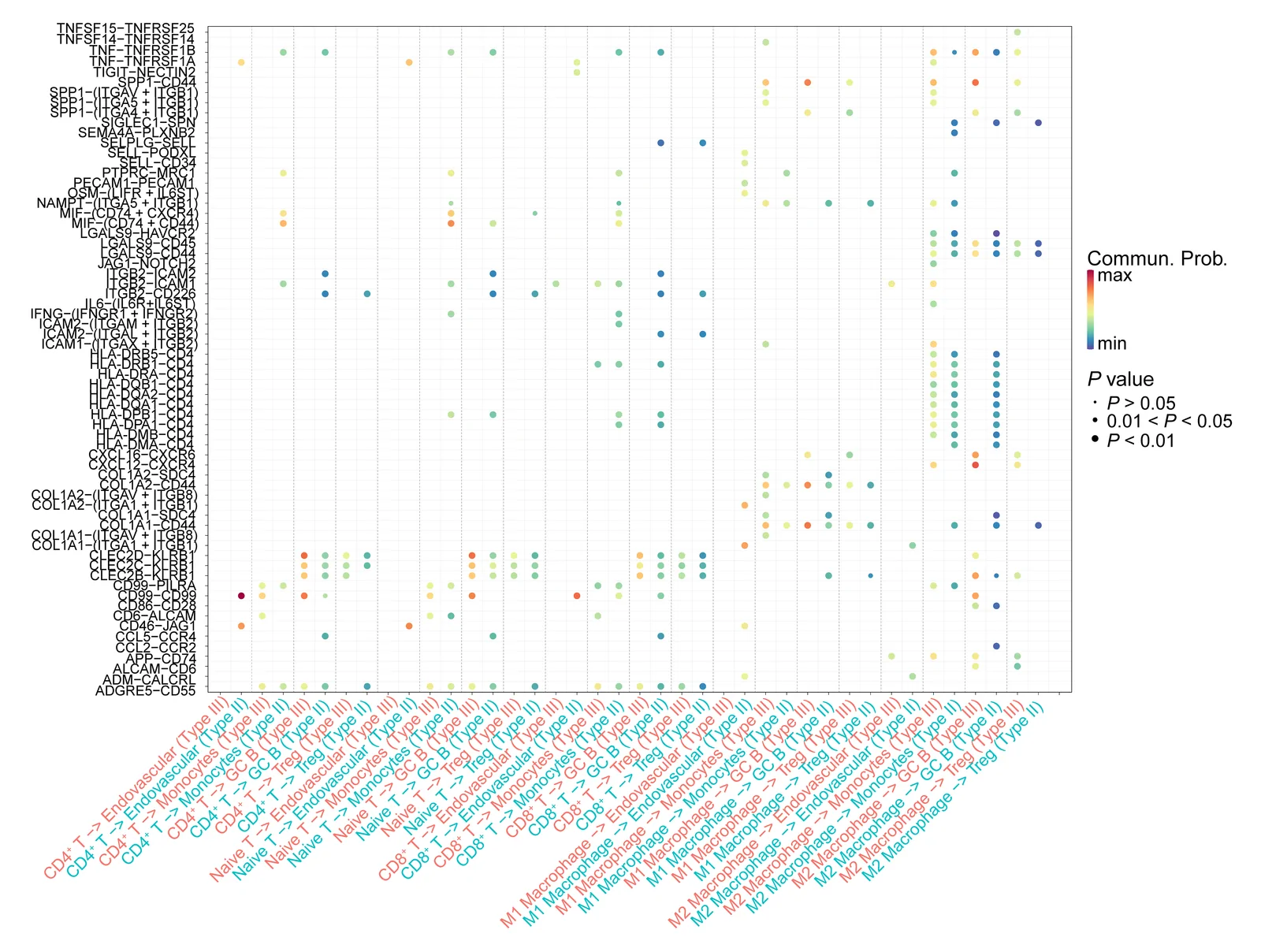
Ligand-receptor interactions between distinct lymphoid and myeloid subsets in Type III versus Type II POFD. Overview of representative ligand-receptor interactions between different subsets of lymphoid (CD4^+^ and CD8^+^ T cell, Naïve T cell, GC B cell, and Tregs) and myeloid (M1 and M2 type macrophage, monocytes, mast cells, DCs, and osteoclast) in Type III versus Type II POFD lesions.

## 4 Discussion

POFD has traditionally been conceptualized as a mosaic disorder driven by somatic *GNAS* mutations that constitutively activate Gsα signaling (1, 4, 12, 64, 65). Evidence that immunocytes govern physiological skeletal turnover strongly implies that mesenchyme-extrinsic immune forces actively shape lesional biology (66–69). In the current study, we employed scRNA-Seq to construct an immune-focused cartography of maxillary POFD across three histological subtypes. Through interrogation of 24,567 individual transcriptomes, we identified subtype-specific differences in lymphoid and myeloid composition: CD8^+^ T lymphocytes and germinal-center B lymphocytes were more abundant in Type III lesions, whereas CD4^+^ helper T cells were more prevalent in Type I. M1 macrophages were enriched in Type II and Type III, and dendritic cells appeared exclusively in Types II and III. These compositional differences were accompanied by distinct ligand-receptor interactome architectures and pathway activation patterns.

Prior scRNA-Seq investigations of fibrous dysplasia have predominantly centered on osteoprogenitor populations (12, 70–74), whereas hematopoietic constituents were frequently excluded. Our finding that adaptive immune elements account for up to 10.9% of cellular biomass in Type III lesions represents a novel discovery. This divergence likely reflects, at least in part, the heightened leukocytic infiltration characteristic of maxillofacial sites relative to appendicular regions (68, 75–79). Earlier reports attributed aberrant CXCL8 expression exclusively to diseased osseous parenchyma (80, 81); conversely, our data localize this chemokine’s production to M1 macrophages and monocytes, highlighting the necessity of resolving cellular provenance when assigning cytokine roles.

Our data indicate that CD4^+^ helper lymphocytes are most abundant in Type I lesions (51.1% of lymphoid cells) and less represented in Types II and III. We speculate that this quantitative difference may reflect subtype-specific immunoregulatory activity. Murine models demonstrate that CD4^+^ T-cell-derived IL-4 attenuates osteoblastic expansion through STAT6-mediated Socs3 upregulation (82–86). The high abundance of LTB-positive CD4^+^ subsets in Type I lesions (**Figure 3B**) may sustain LTβR-mediated paracrine signaling within the mesenchymal compartment. Conversely, the accrual of cytotoxic CD8^+^ lymphocytes in Types II and III (**Figure 3B**) suggests adoption of a cytolytic phenotype. We speculate that CCL3 and CXCL2—identified as network hubs in Type III myeloid populations (**Figure 2D**)—may form a self-reinforcing circuit that entrenches pro-inflammatory signaling, though this hypothesis requires functional validation.

Germinal-center B lymphocytes were absent in Type I, minor in Type II, and prominent in Type III (~29.2% of lymphoid cells) (**Figure 3B**). FTH1 and PABPC1—discriminative transcripts for this subset (**Figure 3E**)—specify an iron-storage protein and an mRNA-stabilizing factor linked to osteoclastogenesis inhibition (87–90). We speculate that GC B-cell-mediated iron sequestration could limit osteoclastic activity, though this mechanism remains hypothetical. Notably, osteoclasts constituted 35.4% of myeloid cells in Type I but were scarce in Types II (1.0%) and III (4.4%) (**Figure 3D**). We cannot exclude the possibility that this striking difference reflects inter-sample heterogeneity rather than a true biological distinction between subtypes. Alternatively, it may reflect the intrinsic biology of each histological category: Type I lesions, rich in fibrous connective tissue, may require active bone resorption for remodeling, whereas the more mineralized Types II and III may exhibit reduced osteoclast demand. The precise role of osteoclasts in each POFD subtype remains undefined and warrants dedicated investigation.

Classically activated M1 macrophages were enriched in Type II (25.7%) and Type III (15.3%) compared with Type I (9.9%) (**Figure 3D**). These cells exhibited transcriptomic enrichment for oxidative-phosphorylation genes, recapitulating the bioenergetic phenotype of pro-inflammatory macrophages (91–95). We speculate that IL1B, CXCL8, and CCL2/3/4 discharge (**Figures 2D**, **3I, J**) may mobilize monocytic effectors. The emergence of dendritic cells bearing B-lymphoid signatures (CD79A, JCHAIN, MS4A1) in Types II and III (**Figure 3F**) implies a plasmacytoid phenotype. We assume that these cells may attempt to present mutant Gsα-derived neoantigens to CD4^+^ T lymphocytes, though this immunoregulatory effort appears insufficient to constrain lesional growth.

Notably, despite employing a comprehensive panel of eight established hematopoietic progenitor cell (HPC) markers (THY1, CD49f, CD34, EPCR, CD135, CD117, CD45RA, and CD38) (96–100), we did not identify a distinct HPC population in our POFD maxillary lesion scRNA-seq dataset. This absence is consistent with the well-documented replacement of hematopoietic marrow by fibro-osseous tissue in FD (101, 102), the minimal baseline hematopoietic activity of adult craniofacial bones (103, 104), and the *GNAS* mutation–induced disruption of the BMSC-derived hematopoietic niche (105–107). Supporting the critical role of the skeletal microenvironment in hematopoietic maintenance, Shen et al. (2021) (108) demonstrated that peri-arteriolar osteolectin-expressing LEPR^+^ stromal cells constitute a mechanosensitive niche for lymphoid progenitors via SCF secretion, whereas Zhang et al. (2025) (109) showed that pathological bone remodeling in disuse osteoporosis drives the expansion of myeloid-biased HSPC subsets with preleukemic epigenetic and transcriptional programs. Collectively, these findings underscore that POFD craniofacial lesions represent a profoundly altered microenvironment in which the normal osteo-hematopoietic axis is fundamentally compromised.

This study has important limitations. The foremost constraint is the restricted sample size (n = 1 per subtype), which precludes robust population-level inference. The observed differences may reflect individual variation rather than true subtype-specific biology. Accordingly, these results should be considered preliminary findings, and readers are urged to interpret them with caution. We are planning to recruit a larger, multi-center POFD cohort to validate these observations. Second, collagenase-based tissue disaggregation may elicit artifactual stress-inducible transcriptional programs. In future studies, it is necessary to jointly utilize scRNA-Seq and the subcellular-resolution spatial transcriptomic platform to investigate the immune regulatory mechanisms underlying different POFD subtypes. Third, our cartography is restricted to the craniofacial skeleton; other anatomical sites may exhibit divergent immune architectures. Fourth, it should be noted that the single-cell RNA 3’-end sequencing approach employed in this study does not permit the detection of *GNAS* coding variants (e.g., c.601C>T [p.Arg201Cys] and c.602G>A [p.Arg201His]) due to the limited transcript coverage at the 3’ terminus. Consequently, we were unable to determine which specific cell lineages harbor the activating *GNAS* mutation in our POFD craniofacial lesions. Future work from our group will leverage single-cell long-read sequencing platforms to simultaneously profile transcriptomic states and *GNAS* mutation status at the single-cell level, thereby directly addressing the lineage-specific consequences of *GNAS* mosaicism in POFD pathogenesis. Fifth, the candidate biomarkers and potential therapeutic targets identified herein require validation in larger, independent cohorts and functional studies before any clinical application can be considered. Finally, all mechanistic interpretations proposed in this study are hypotheses derived from computational analyses and literature correlation, not from experimental perturbation. In future studies, direct *in vitro* and *in vivo* manipulation is essential to establish causality.

## 5 Conclusions

By deploying single-cell transcriptomics across three POFD histological subtypes, we reveal that lesional immune composition differs markedly among connective-tissue-predominant, cancellous-bone-predominant, and sclerotic-bone-predominant categories. The subtype-specific markers (*LTB*, *TXNIP*, *FTH1*, *PABPC1*, *CXCL8*, and *IL1B*) and communication architectures identified herein offer candidate biomarkers for diagnostic stratification and nominate hypothetical therapeutic targets. However, these candidates require rigorous validation before clinical translation. Our data emphasize that POFD lesions are immunologically heterogeneous, providing a foundation for future mechanism-based studies.

## Data Availability

The raw data have been deposited to GEO repository with the accession number GSE346343.

## Glossary

ACP5: acid phosphatase 5, tartrate resistant
ACTG1: actin gamma 1
ADGRE5: adhesion G protein-coupled receptor E5
ANXA2: annexin A2
BMP-2: bone morphogenetic protein 2
BMSC: bone marrow stromal cell
CALM2: calmodulin 2
cAMP: cyclic AMP
CCL2: C-C motif chemokine ligand 2
CLEC2D: C-type lectin domain family 2 member D
CXCL8: C-X-C motif chemokine ligand 8
CXCL12: C-X-C motif chemokine ligand 12
CXCR4: C-X-C motif chemokine receptor 4
CTSK: cathepsin K
DC: dendritic cell
DEG: differentially expressed gene
DNAJA1: DnaJ heat shock protein family member A1
EEF2: eukaryotic translation elongation factor 2
EIF4A1: eukaryotic translation initiation factor 4A1
FTH1: ferritin heavy chain 1
GC: germinal-center
GNAS: GNAS complex locus
GO: gene ontology
GPCR: G protein-coupled receptor
GZMB: granzyme B
HNRNPA1: heterogeneous nuclear ribonucleoprotein A1
HSPA1B: heat shock protein family A (Hsp70) member 1B
HSPA6: heat shock protein family A (Hsp70) member 6
HSPC: hematopoietic stem and progenitor cell
HSPE1: heat shock protein family E (Hsp10) member 1
IL1B: interleukin 1 beta
IL4R: interleukin 4 receptor
KLRB1: killer cell lectin like receptor B1
LEPR: leptin receptor
LTB: lymphotoxin beta
MAS: McCune-Albright syndrome
MT1X: metallothionein 1X
MX1: MX dynamin like GTPase 1
NF-κB: nuclear factor kappa-B
ODF: osteoclast differentiation factor
PABPC1: poly(A) binding protein cytoplasmic 1
POFD: polyostotic fibrous dysplasia
PPI: protein-protein interaction
RACK1: receptor for activated C kinase 1
RANKL: receptor activator of NF-κB ligand
S100A8: S100 calcium binding protein A8
SCF: stem cell factor
scRNA-Seq: single-cell RNA sequencing
TNF-α: tumor necrosis factor-alpha
TNFRSF4: TNF receptor superfamily member 4
TRBC2: T cell receptor beta constant 2
Treg: regulatory T cells
TXNIP: thioredoxin interacting protein
XAF1: XIAP associated factor 1
