# Supplementary material for: Single-cell landscape of lymphoid and myeloid heterogeneity across type-stratified craniofacial lesions in polyostotic fibrous dysplasia
Source: Front Immunol. 2026 Sep 28;17:1906418. doi: 10.3389/fimmu.2026.1906418 (PMC13655417; doi:10.3389/fimmu.2026.1906418)
Supplement: Supplementary file 1 [file DataSheet1.pdf]

Supplementary figures to:

Single-Cell Landscape of Lymphoid and Myeloid Heterogeneity Across Type-Stratified Craniofacial Lesions in Polyostotic Fibrous Dysplasia

Hui-Chuan Duan[1†], Xia Liang[2†], De-Cheng Wang[3], Jie Yuan[1*], Fan Yang[4,5*]

[1] Department of Plastic and Reconstructive Surgery, Shanghai 9th People's Hospital, Shanghai Jiao Tong University School of Medicine, Shanghai, 200011, China

[2] Department of Addiction Medicine, Anding Hospital Wuhu Branch (Wuhu Fourth People's Hospital), Wuhu, 242407, China

[3] The Research Center for Lin He Academician New Medicine, Institutes for Shanghai Pudong Decoding Life, Shanghai, 200030, China

[4] Lishui Key Laboratory of Brain Health and Severe Brain Disorders, Lishui Second People's Hospital, Wenzhou Medical University, Lishui, 323000, China

[5] Bio-X Institutes, Key Laboratory for the Genetics of Developmental and Neuropsychiatric Disorders, Ministry of Education, Shanghai Jiao Tong University, Shanghai, 200030, China

[†] These authors contributed equally to this work.

[*]**Corresponding author**:

Fan Yang, Ph.D., Associate Investigator. Email: yangfan@sibs.ac.cn

Address: No.69, Beihuan Road, Liandu District, Lishui Key Laboratory of Brain Health and Severe Brain Disorders, Lishui Second People's Hospital, Wenzhou Medical University, Lishui, 323000, China.

[*]**Co-corresponding author**: Jie Yuan, M.D., Ph.D., email: Jaman1@126.com

Running title: Immune Landscapes Across POFD Subtypes

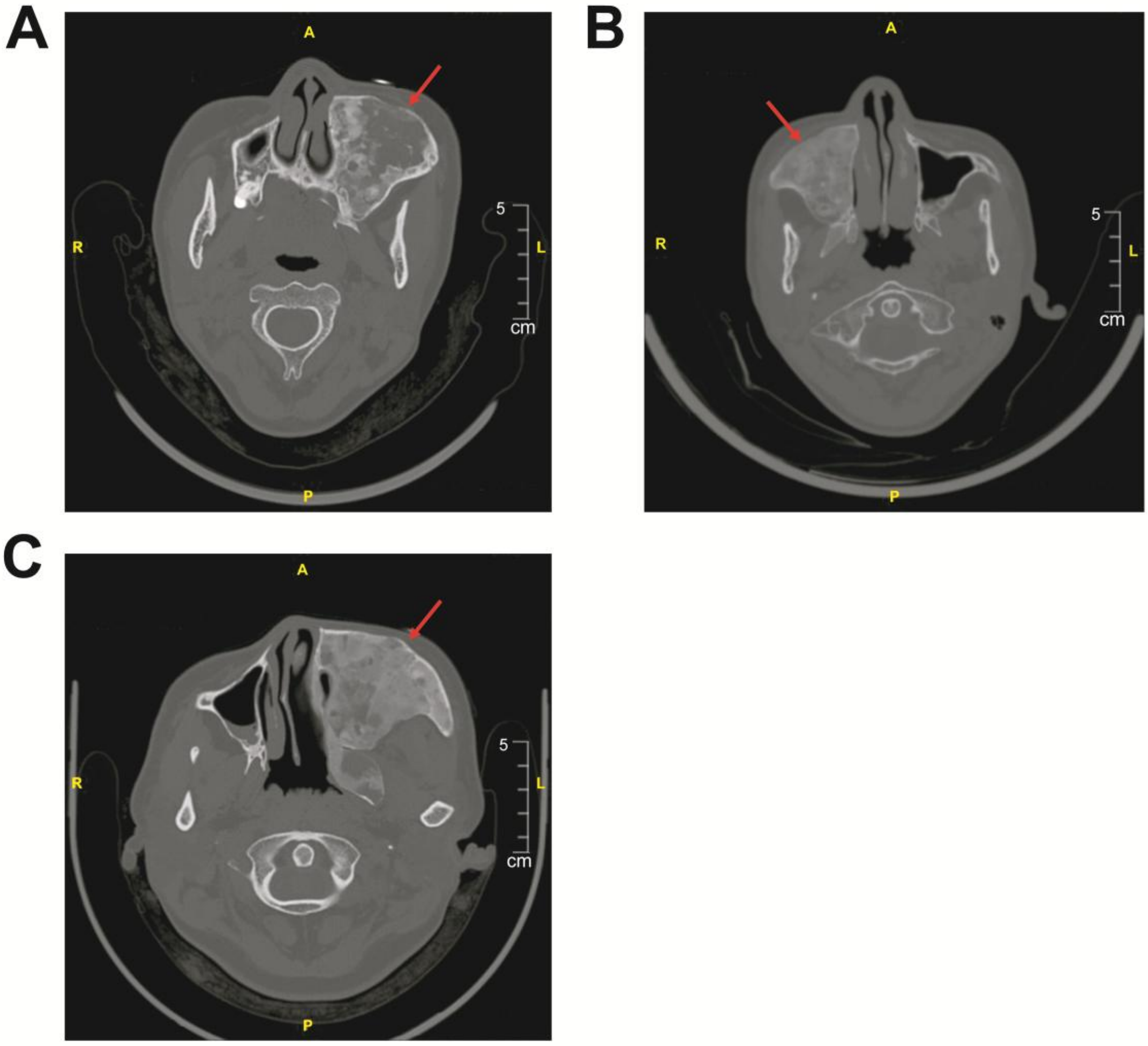

Supplementary Figure 1. CT imaging characteristics of three POFD histological subtypes. (**A**) Type I (connective-tissue predominant): Round or oval radiolucent lesions of variable size resembling bone cysts, with round or oval radiolucent lesions of variable size; note the bubbly appearance in a subset of lesions. (**B**) Type II (cancellous-bone predominant): Classic ground-glass appearance with effacement of normal trabecular architecture. (**C**) Type III (sclerotic-bone predominant): Markedly hyperdense relative to Types I and II, with thickened and tortuous trabeculae; some lesions exhibit peripheral ground-glass change and sclerotic rims. R, right; L, left; A, anterior; P, posterior. Red arrow indicates the lesions of different POFD subtypes.

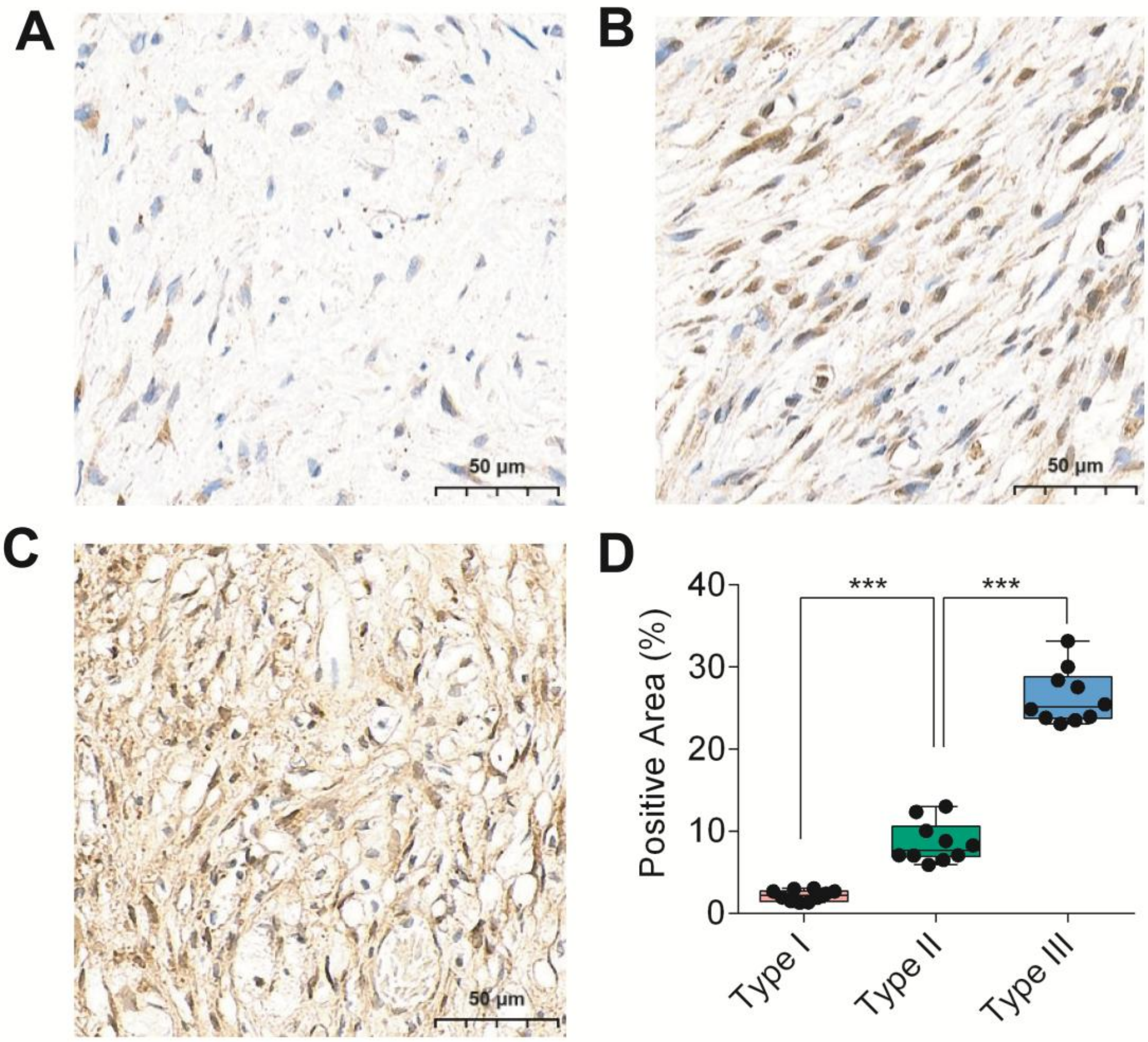

Supplementary Figure 2. Immunohistochemical validation of IL1B expression across craniofacial lesions from the three POFD subtypes. (**A-C**) Representative IHC images showing IL1B expression in independent Type I (**A**), Type II (**B**), and Type III (**C**) POFD tissue sections. Orange-yellow signals indicate IL1B positivity; blue signals indicate hematoxylin-counterstained nuclei. Scale bar, 50 μm; magnification, 400×. (**D**) Box plot quantifying IL1B expression as positive area (%) across Type I, Type II, and Type III lesions. Ten random fields per subtype were analyzed. Group comparisons were performed using independent-samples t-tests. ***: $P < 0.001$.

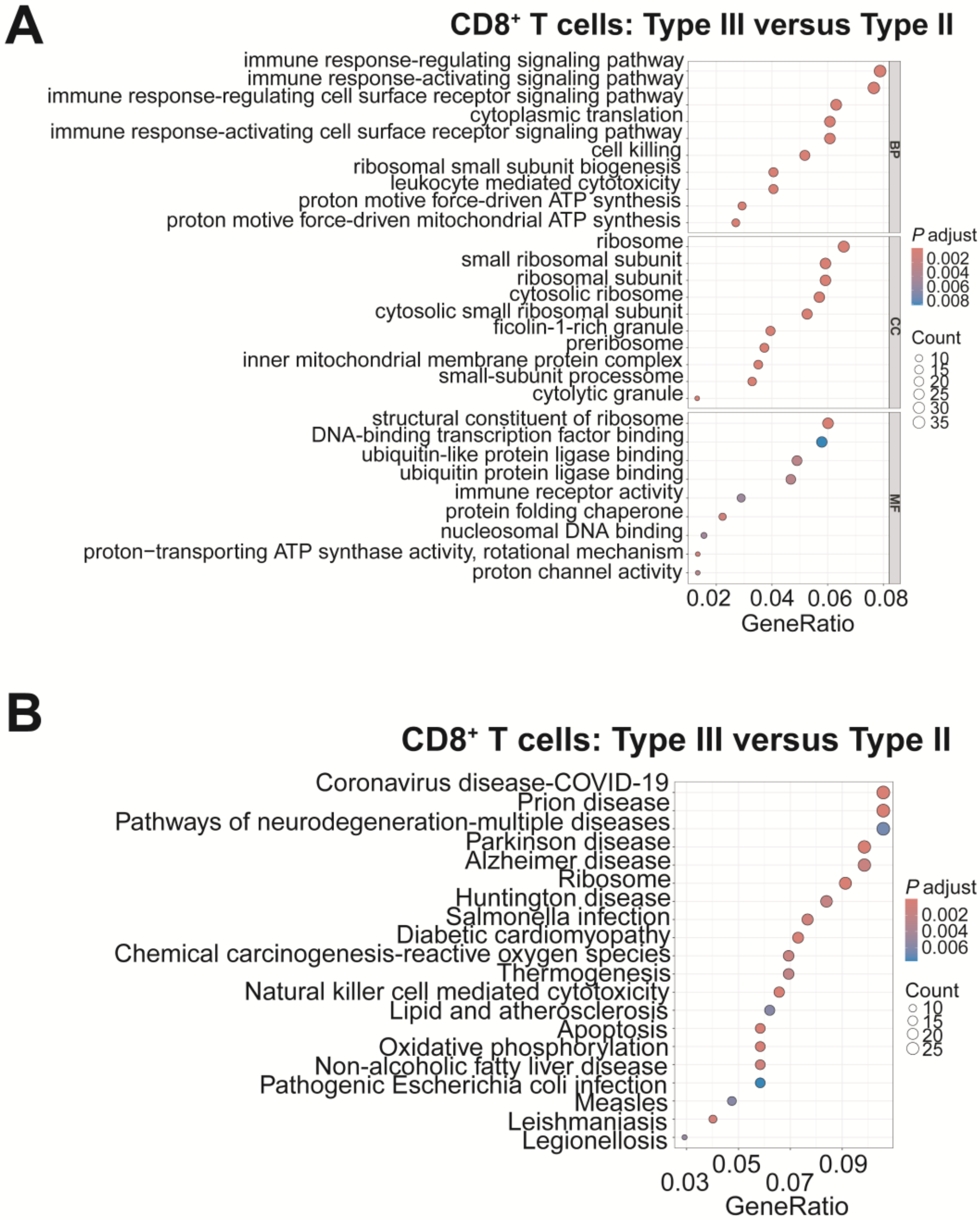

Supplementary Figure 3. Pathway enrichment analysis of CD8⁺ T cells DEGs in Type III versus Type II lesions. (**A**) GO enrichment analysis of CD8⁺ T cells DEGs in Type III versus Type II lesions. BP: biological process; CC: cellular compartment; MF: molecular function. (**B**) KEGG pathway enrichment analysis of CD8⁺ T cells DEGs in Type III versus Type II lesions.

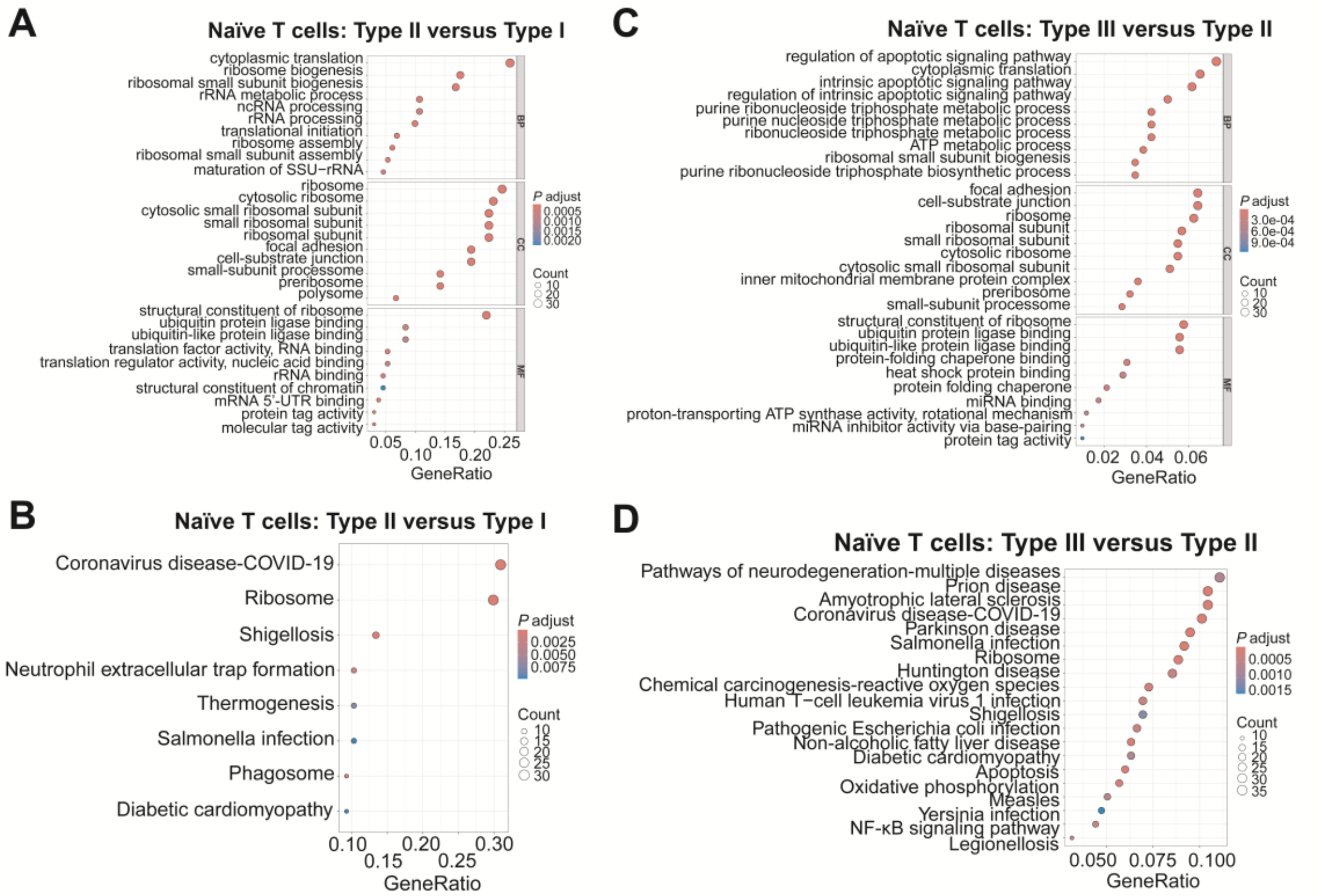

Supplementary Figure 4. Pathway enrichment analysis of Naïve T cells DEGs in the three POFD subtypes. (**A**) GO enrichment analysis of Naïve T cells DEGs in Type II versus Type I lesions. (**B**) KEGG pathway enrichment analysis of Naïve T cells DEGs in Type II versus Type I lesions. (**C**) GO enrichment analysis of Naïve T cells DEGs in Type III versus Type II lesions. (**D**) KEGG pathway enrichment analysis of Naïve T cells DEGs in Type III versus Type II lesions. (**A**) and (**C**) BP: biological process; CC: cellular compartment; MF: molecular function.

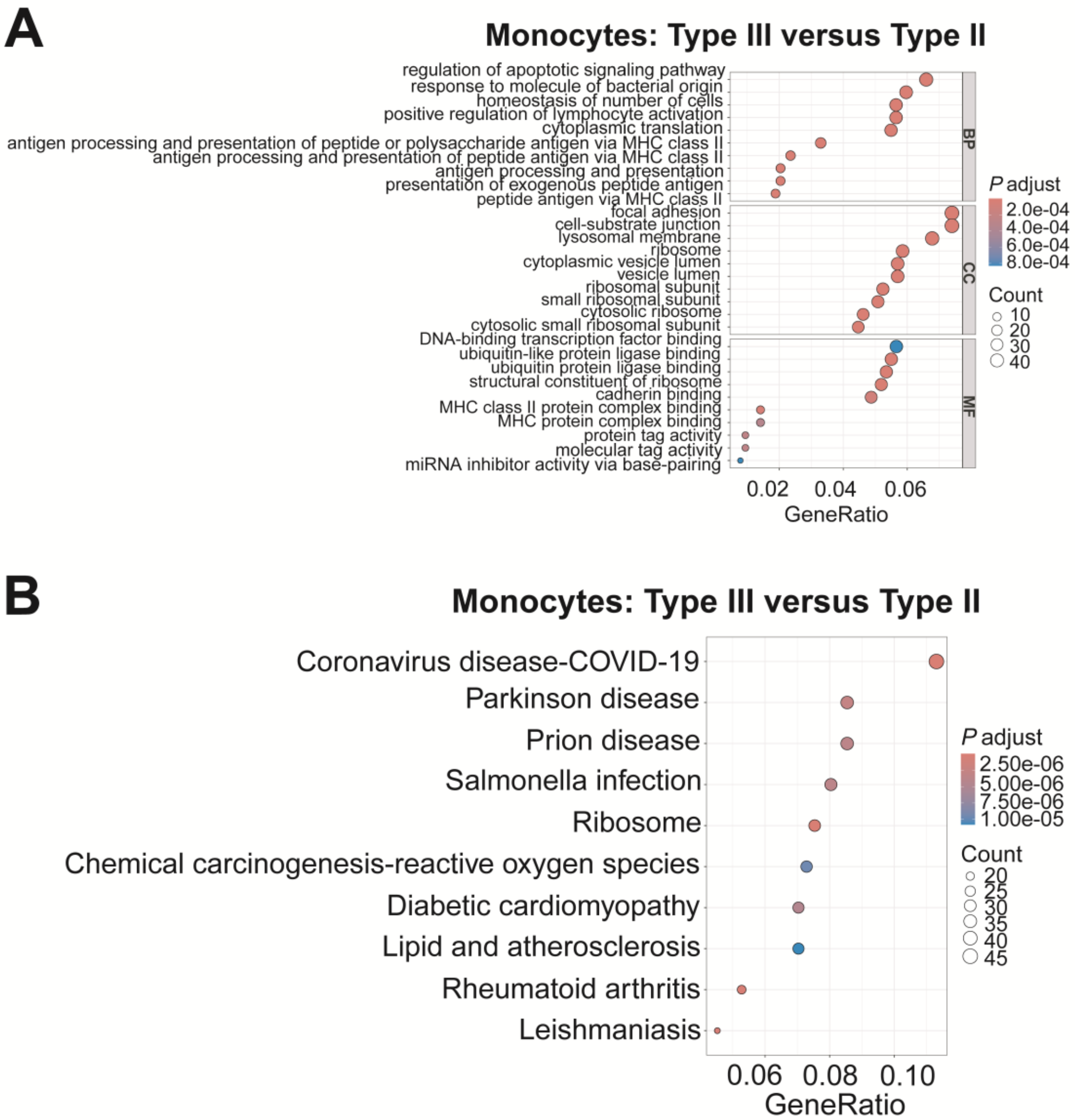

Supplementary Figure 5. Pathway enrichment analysis of monocytes DEGs in Type III versus Type II lesions. (**A**) GO enrichment analysis of monocytes DEGs in Type III versus Type II lesions. BP: biological process; CC: cellular compartment; MF: molecular function. (**B**) KEGG pathway enrichment analysis of monocytes DEGs in Type III versus Type II lesions.

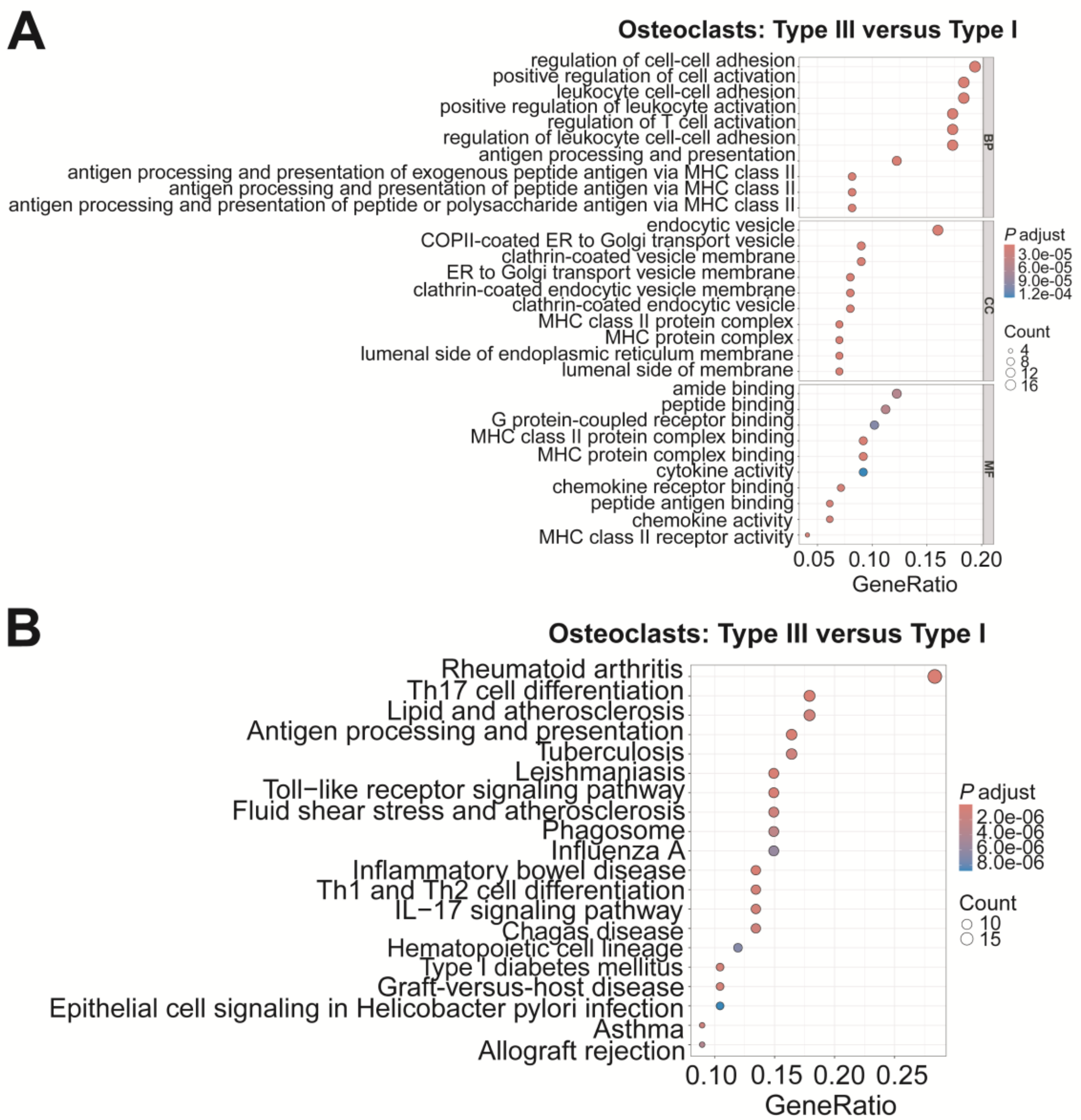

Supplementary Figure 6. Pathway enrichment analysis of osteoclasts DEGs in Type III versus Type I lesions. (**A**) GO enrichment analysis of osteoclasts DEGs in Type III versus Type I lesions. BP: biological process; CC: cellular compartment; MF: molecular function. (**B**) KEGG pathway enrichment analysis of osteoclasts DEGs in Type III versus Type I lesions.
